# Expression of MHC II in DRG neurons attenuates paclitaxel-induced cold hypersensitivity in male and female mice

**DOI:** 10.1371/journal.pone.0298396

**Published:** 2024-02-08

**Authors:** Emily E. Whitaker, Neal E. Mecum, Riley C. Cott, Diana J. Goode

**Affiliations:** Department of Biomedical Sciences, College of Osteopathic Medicine, University of New England, Biddeford, Maine, United States of America; UCSD: University of California San Diego, UNITED STATES

## Abstract

Chemotherapy is often a life-saving treatment, but the development of intractable pain caused by chemotherapy-induced peripheral neuropathy (CIPN) is a major dose-limiting toxicity that restricts cancer survival rates. Recent reports demonstrate that paclitaxel (PTX) robustly increases anti-inflammatory CD4^+^ T cells in the dorsal root ganglion (DRG), and that T cells and anti-inflammatory cytokines are protective against CIPN. However, the mechanism by which CD4^+^ T cells are activated, and the extent cytokines released by CD4^+^ T cells target DRG neurons are unknown. Here, we are the first to detect major histocompatibility complex II (MHCII) protein in mouse DRG neurons and to find CD4^+^ T cells breaching the satellite glial cell barrier to be in close proximity to neurons, together suggesting CD4^+^ T cell activation and targeted cytokine release. MHCII protein is primarily expressed in small nociceptive neurons in male and female mouse DRG but increased after PTX in small nociceptive neurons in only female DRG. Reducing one copy of MHCII in small nociceptive neurons decreased anti-inflammatory IL-10 and IL-4 producing CD4^+^ T cells in naïve male DRG and increased their hypersensitivity to cold. Administration of PTX to male and female mice that lacked one copy of MHCII in nociceptive neurons decreased anti-inflammatory CD4^+^ T cells in the DRG and increased the severity of PTX-induced cold hypersensitivity. Collectively, our results demonstrate expression of MHCII protein in mouse DRG neurons, which modulates cytokine producing CD4^+^ T cells in the DRG and attenuates cold hypersensitivity during homeostasis and after PTX treatment.

## Introduction

Paclitaxel (PTX) is an anti-neoplastic drug commonly used to treat breast, lung, and ovarian cancer [1]. However, this life-saving cancer treatment is often dose-limiting as more than 60% of patients develop painful chemotherapy-induced peripheral neuropathy (CIPN) [2], including those taking PTX [3]. Endothelial cells encapsulating dorsal root ganglia (DRG) have large fenestrations [4] with an altered repertoire of tight junction proteins [5] allowing PTX to enter the DRG and to bind to toll-like receptor 4 (TLR4) expressed on macrophages and DRG neurons [6]. As a result, PTX increases pro-inflammatory cytokines (TNF-⍺ and IL-6) in the DRG [7, 8]. These cytokines along with PTX act on DRG neurons inducing hyperexcitability [9–11] and neurotoxicity [12, 13], which manifests as pain, tingling, and numbness in a stocking and glove distribution [14]. Recently, T cells and anti-inflammatory cytokines (IL-4 and IL-10) have been shown to suppress CIPN [15–17]; however, the underlying mechanism contributing to the resolution of CIPN remains elusive, which is evidenced by limited treatment options.

While treatment with PTX robustly increases anti-inflammatory CD4^+^ T cells in female mouse DRG [18], the mechanism by which this occurs is unclear. It is thought that communication between neurons and CD4^+^ T cells primarily occurs through the release of soluble mediators as neurons express cytokine receptors [19] while CD4^+^ T cells express receptors for neurotransmitters [20]. Pro-inflammatory CD4^+^ T cell subsets (Th1 and Th17) secrete cytokines that sensitize neurons in the DRG and spinal cord to increase nociceptive-signaling [21, 22] while anti-inflammatory subsets (FoxP3 Tregs and Th2) secrete IL-10 and IL-4, which suppress activity in sensitized nociceptors and reduce neuronal hyperexcitability [15], respectively. Although CD4^+^ T cell–neuron communication through soluble mediators has been more extensively studied, published DRG neuron RNA seq mouse and human datasets [23, 24] indicate direct cell-cell interaction is possible. By mining these single cell RNA seq data sets, we found that DRG neurons express major histocompatibility complex II (MHCII) transcripts [25, 26], but there are no reports that show MHCII protein or function in DRG neurons.

MHCII is traditionally thought to be constitutively expressed on antigen-presenting cells (APCs) and induced by inflammation on some non-APCs, including endothelial, epithelial, and glial cells [27]. In contrast to these non-APCs, human neural stem cells (hNSCs) and neural progenitor cells within embryo spinal cord and DRG constitutively express MHCII protein prior to the development of the adaptive immune response [28]. Differentiating hNSCs to neurons in vitro increased MHCII, and the addition of inflammatory stimuli (IFN-γ) further elevated expression [28]. Based on this finding, we wanted to investigate whether terminally differentiated neurons expressed MHCII in vivo as this would provide insight into the mechanism by which CD4^+^ T cells contribute to pain and neurological diseases.

While DRG neurons are wrapped with satellite glial cells (SGCs), natural gaps in the glial envelope can occur [29]. Furthermore, sciatic nerve injury and nerve transection models demonstrate that immune cells can breach the SGC and lie directly against the neuron [30], suggesting direct neuron-immune communication. In our present study, we found that CD4^+^ T cells can breach the SGC barrier during homeostasis and after PTX treatment, and that DRG neurons express functional surface-bound MHCII protein. Moreover, neuronal MHCII represents a novel target to suppress CIPN, which could be exploited for therapeutic intervention against not only pain but potentially autoimmunity and neurological diseases.

## Results

### CD4^+^ T cells breach the SGC barrier in mouse DRG

**Fig 1:**
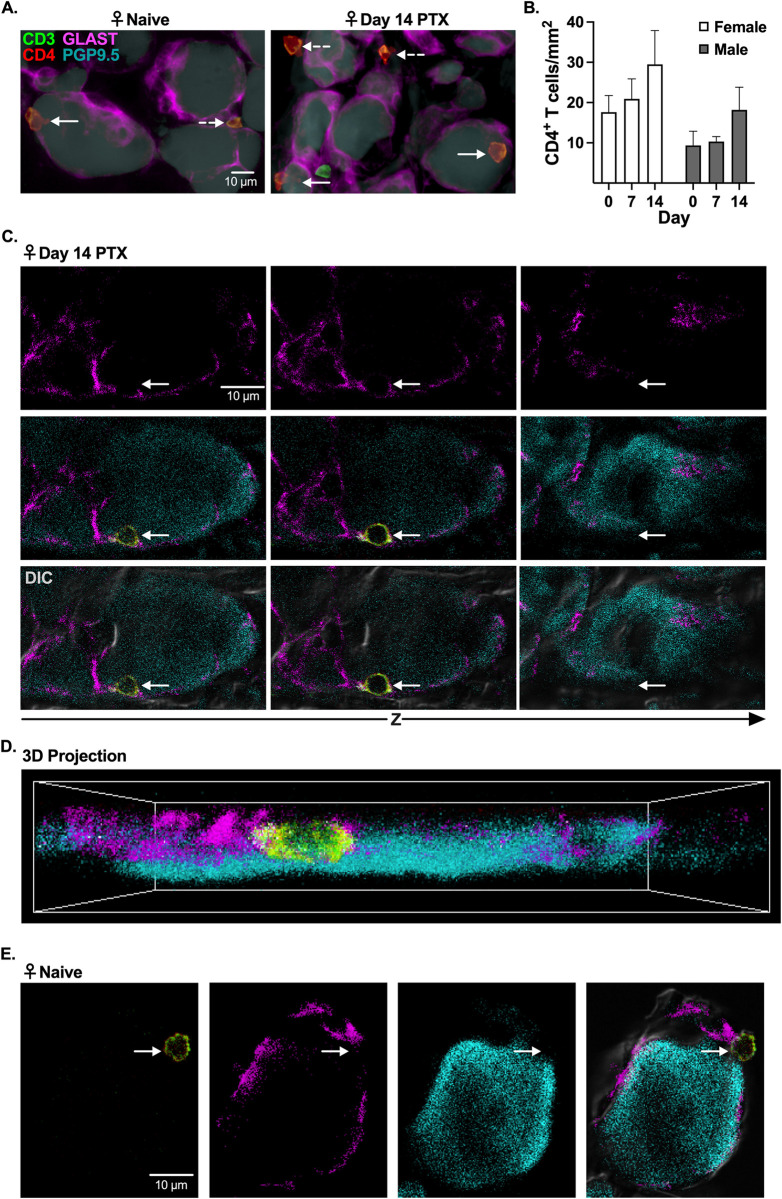
CD4^+^ T cells breach SGC barrier surrounding neurons in DRG tissue from naïve and PTX-treated mice. **(A-E)** Immunohistochemical (IHC) staining of neurons (PGP9.5: cyan), SGCs (GLAST: magenta), and T cells (CD3: green, CD4: red) in lumbar 4 (L4) DRG tissue from naïve and day 14 PTX-treated female and male mice. **(A)** Representative widefield fluorescence of CD4^+^ T cells (CD3^+^ CD4^+^) and neurons (PGP9.5^+^) in close proximity (solid arrow = absence of GLAST; dashed arrow = occluded by GLAST) in DRG tissue from naïve and PTX-treated female mice. **(B)** Quantification of the number of CD4^+^ T cells per mm^2^ of DRG tissue in female and male naïve and PTX-treated mice (day 7 and 14) (n = 8). **(C)** Differential inference contrast (DIC), fluorescence, and merged confocal Z-slice images of a CD4^+^ T cell between the SGC barrier and a PGP9.5^+^ DRG neuron from a PTX-treated female mouse. **(D)** 3D projection of a CD4^+^ T cell shown in **(C, S1 Fig in S1 File and S1 Video)**. **(E)** Maximum projection images of a CD4^+^ T cell that breached the SGC barrier surrounding a neuron in DRG tissue from a naïve female mouse.

We recently demonstrated that CD4^+^ T cells in the DRG primarily produce anti-inflammatory cytokines [18]. Due to their short half-life, cytokine release typically occurs in close proximity to the target cell; however, the extent cytokines released by CD4^+^ T cells target DRG neurons in vivo is unknown. Therefore, initial fluorescent imaging experiments were designed to visualize the location of CD4^+^ T cells within DRG tissue. We found that CD4^+^ T cells were consistently present in female and male DRG from naïve and day 14 PTX-treated mice and frequently clustered in close proximity to neurons. **(Fig 1A**, n = 8**)**. Although not significant, there tended to be more CD4^+^ T cells in the DRG of day 14 PTX-treated female (17.61 ± 4.13 for naïve and 29.47 ± 8.39 CD4^+^ T cells 14 days post PTX, p = 0.1927, n = 8) and male (9.34 ± 3.56 for naïve and 18.19 ± 5.62 CD4^+^ T cells 14 days post PTX, p = 0.3788, n = 8) mice than naïve mice (**Fig 1B**). Confocal microscopy was required to demonstrate that CD4^+^ T cells colocalized with neurons in the absence of SGC markers (GLAST/FABP7) in DRG tissue from naïve and day 14 PTX-treated female mice **(Fig 1C–1E, S1 Video, S2 Fig in S1 File)**. Z-stacks of DRG tissue confirmed that CD4^+^ T cells were located between the border (identified by differential interference contrast microscopy) of DRG neurons and SGCs **(Fig 1C**, **S2A Fig in S1 File)**. Our results demonstrate that CD4^+^ T cells can breach the SGCs barrier, suggesting CD4^+^ T cell–DRG neuron communication can occur through both cell-cell contact and the release of soluble mediators.

Expression of immune molecules on DRG neurons could facilitate communication between CD4^+^ T cells and DRG neurons. Indeed, published RNA seq data sets demonstrate that both human and mouse DRG neurons [25, 31] express MHCII and MHCII-associated genes, which are critical for the activation of CD4^+^ T cells upon cell-cell contact. Consistent with the RNA seq data, which shows expression of RFX1 RNA [25, 31], we detected RFX1 protein, a transcription factor for MHCII, in the nucleus of DRG neurons in male and female mice **(S3 Fig in S1 File)**, further supporting the possibility of MHCII protein in DRG neurons.

### MHCII protein detected in DRG neurons

**Fig 2:**
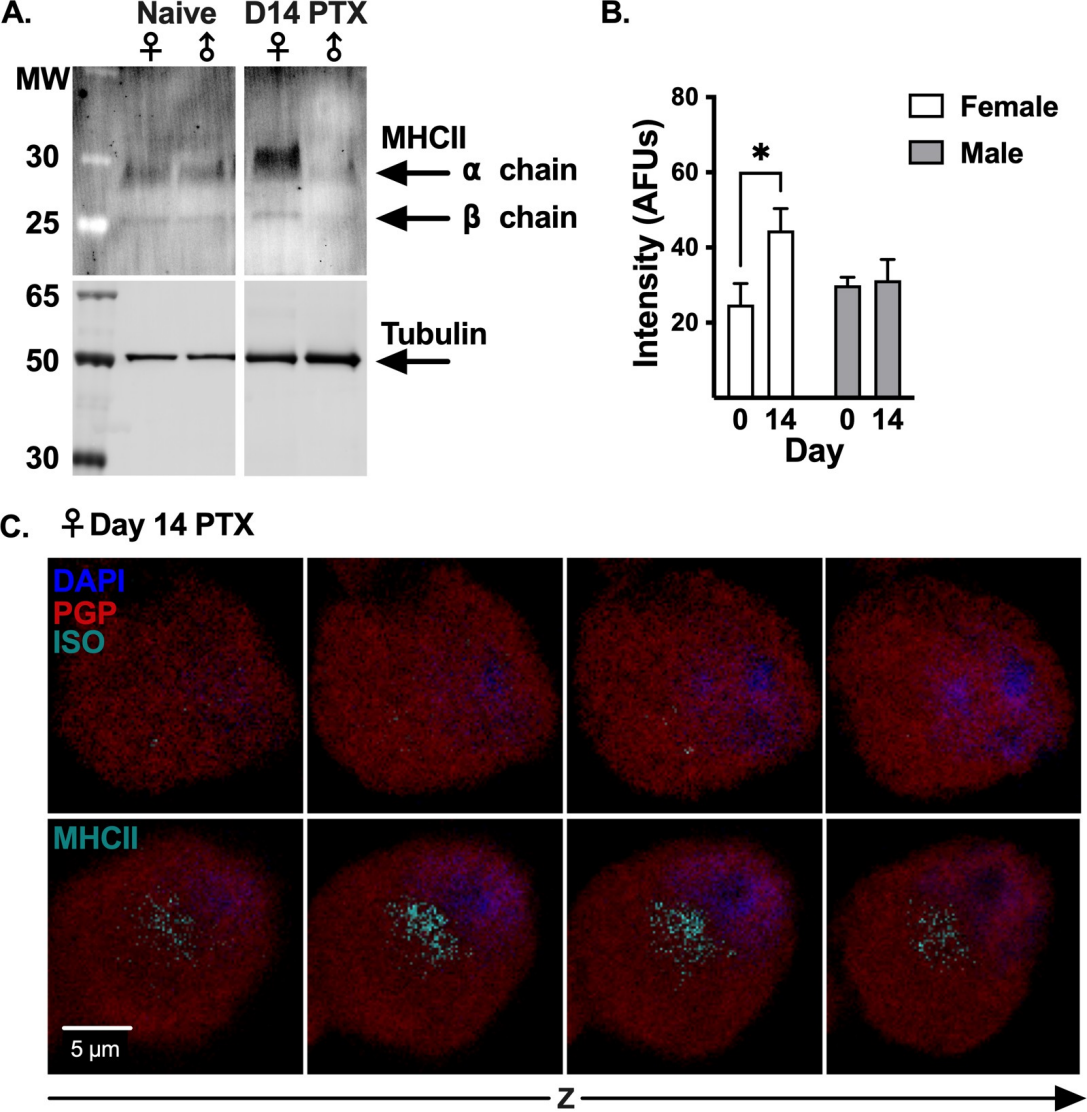
DRG neurons from female and male mice express MHCII protein. **(A)** Western blot of MACS-DRG neuron lysates from naïve and day 14 PTX-treated female and male mice, probed with antibodies against MHCII and beta tubulin (loading control). Irrelevant lanes removed from blot. **(B)** Quantification of MHCII band intensity normalized to tubulin for DRG neuron lysates from naïve and day 14 PTX-treated female (white) and male (gray) mice shown in **(A)**. Statistical significance determined by 2-way ANOVA with Sidak’s multiple comparison test (*p <0.05, n = 4–7). **(C)** Confocal stepwise Z-slices of cultured PGP9.5^+^ DRG neuron (red) from a day 14 PTX-treated female mouse stained with DAPI (blue) and antibody against MHCII or isotype-control antibody (cyan).

Inflammatory stimuli are known to increase the expression of MHCII in immune cells [32]; therefore, mice were injected with PTX to induce inflammation in the DRG. DRG neuron lysates from naïve and day 14 PTX-treated female and male mice were probed with an antibody against MHCII and analyzed by western blot. Consistent with inflammatory stimuli boosting the expression of MHCII, DRG neurons from PTX-treated female mice had almost 2-fold more MHCII protein than DRG neurons from naïve female mice (24.77 ± 5.62 arbitrary fluorescent units (AFUs) from naïve to 44.48 AFUs ± 5.83 14 days post-PTX, normalized to tubulin, p = 0.0276, n = 4–7) **(Fig 2A and 2B)**. In contrast, PTX treatment did not change the amount of MHCII protein detected in DRG neurons from male mice (29.99 ± 2.13 AFUs from naïve to 31.33 AFUs ± 5.52 14 days post-PTX, p = 0.9855, n = 4–7) **(Fig 2A and 2B)**.

Although cell lysates used for western blot primarily consisted of DRG neurons, negative selection by magnetic-activated cell sorting (MACS) does not yield a cell sample that is 100% pure. Therefore, it is possible that the MHCII protein signal detected by western blot represents MHCII from contaminating non-neuronal cells. To verify that DRG neurons express MHCII protein, we visualized MHCII by immunocytochemistry (ICC) of cultured DRG neurons from day 14 PTX-treated female mice, which had the highest expression of MHCII by western blot. Nuclei were labeled with DAPI to eliminate the possibility that MHCII staining was the result of a contaminating non-neuronal MHCII^+^ cell. Z-stack images acquired by confocal microscopy demonstrate MHCII staining through the entire DRG neuron from day 14 PTX-treated female mice compared to the isotype-stained control **(Fig 2C)**, confirming expression of MHCII protein in DRG neurons.

### DRG neurons express surface-MHCII

**Fig 3:**
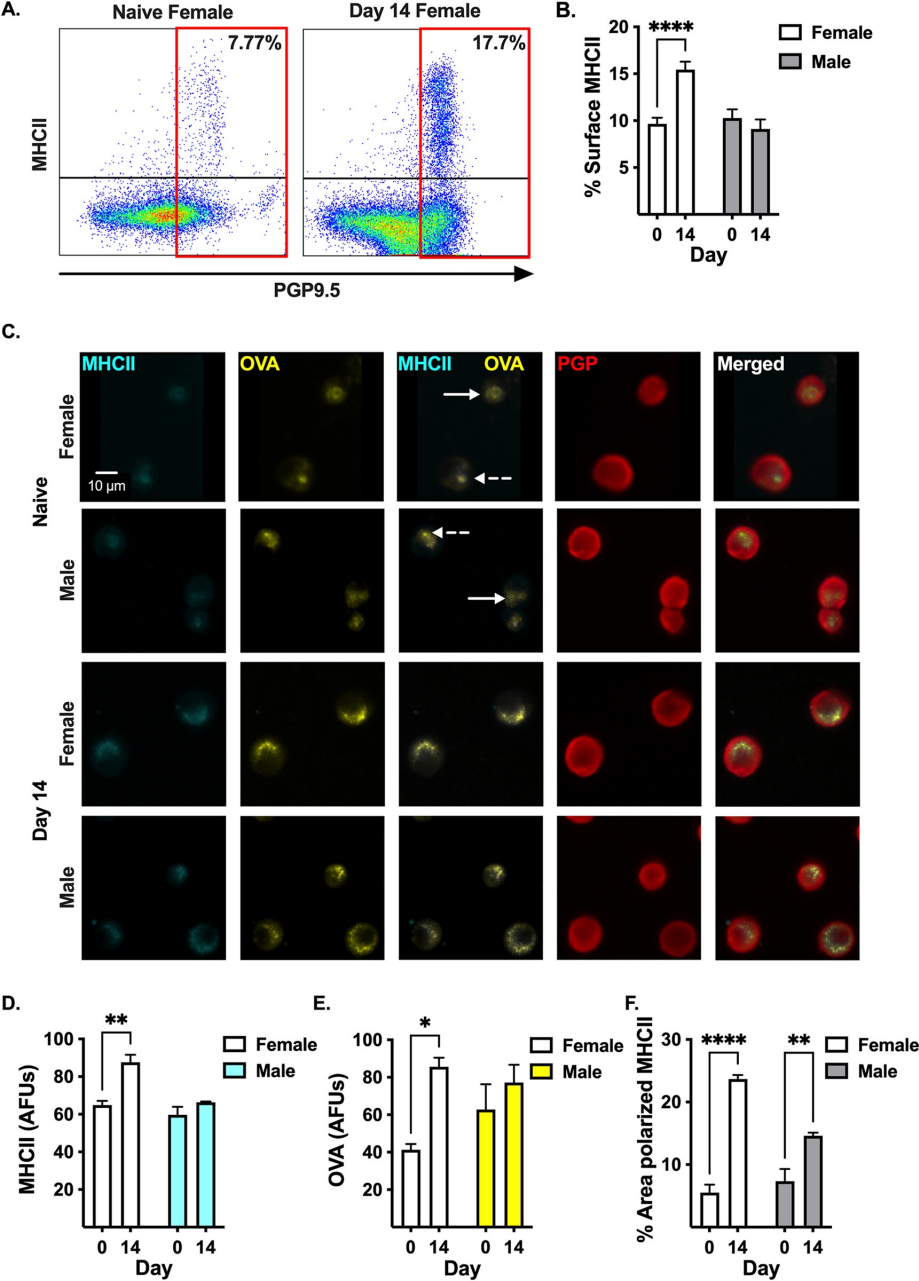
PTX treatment increases surface expression of peptide-bound MHCII on DRG neurons from female mice. **(A)** Representative flow cytometric dot plots of the frequency of DRG neurons expressing surface-MHCII from naïve and day 14 PTX-treated female mice. **(B)** Quantification of the frequency of DRG neurons from female (white bar, n = 9) and male (gray bar, n = 6) mice expressing surface-MHCII shown in **(A)**. **(C)** Representative widefield fluorescence microscopy images of DRG neurons (PGP9.5^+^: red) expressing surface-MHCII (cyan) co-localized with OVA-FITC (yellow) from naïve and day 14 PTX-treated female and male mice. Diffuse (solid arrow) and punctate (dashed arrow) surface-MHCII staining in DRG neurons from naïve female and male mice. Quantification of **(D)** MHCII intensity, **(E)** OVA intensity, and **(F)** percent of neuron area with polarized MHCII from **(C)**. **(D-F)** Statistical significance determined by 2-way ANOVA with Sidak’s multiple comparison test (*p <0.05, **p < 0.01, ****p < 0.0001, n = 3).

MHCII on the cell surface is required to activate CD4^+^ T cells. We performed flow cytometry to determine whether neurons express surface-MHCII. PGP9.5^+^ neurons comprised 42.37% ± 1.79 of total DRG cells **(S4 Fig in S1 File)**, and within the PGP9.5^+^ neuron gate, 9.66% ± 0.64 of cells from naïve female mice expressed surface-MHCII **(Fig 3A and 3B)**. Surface-MHCII on DRG neurons increased to 15.44% ± 0.84 after treatment with PTX (p<0.0001, n = 9) **(Fig 3A and 3B)**, which is consistent with inflammation increasing the half-life of MHCII on the surface of APCs [33]. In agreement with the western blot, surface-MHCII did not increase after PTX on DRG neurons from male mice (10.28% ± 0.93 in naïve and 9.11% ± 1.01 14 days post-PTX, p = 0.6054, n = 6) **(Fig 3B)**. MHCII on the surface of neurons suggests a mechanism by which CD4^+^ T cells could directly communicate with DRG neurons.

CD4^+^ T cells respond to specific peptide bound to surface-MHCII; however, it is unknown if surface-MHCII on neurons can present peptide. To address this question, we cultured DRG neurons from naïve and day 14 PTX-treated female and male mice with FITC-conjugated OVA peptide. We found that PGP9.5^+^ DRG neurons from naïve female and male mice expressed surface-MHCII **(Fig 3C)**, which co-localized with the OVA-FITC signal **(Fig 3C)**, suggesting OVA peptide is bound to surface-MHCII. Of note, some naïve DRG neurons have diffuse OVA-MHCII signal (**Fig 3C, solid white arrow)** while others have punctate (polarized) OVA-MHCII staining **(Fig 3C, dashed white arrow)**. PTX treatment in vivo significantly increased surface-MHCII on cultured neurons compared to neurons from naïve female mice (64.87 ± 2.29 AFUs naïve to 87.59 ± 4.07 AFUs day 14, p = 0.0019, n = 3) **(Fig 3D)**. In contrast, PTX treatment did not change surface-MHCII on cultured neurons from male mice (59.72 ± 4.22 AFUs naïve to 66.35 ± 0.42 AFUs day 14, p = 0.3200, n = 3) (**Fig 3D)**. The intensity of OVA increased >2-fold on neurons from day 14 PTX-treated female mice compared to neurons from naïve female mice (41.30 ± 3.10 AFUs naïve to 85.62 ± 4.80 AFUs day 14, p = 0.0143, n = 3) **(Fig 3E)**. Like surface-MHCII, PTX did not change the intensity of OVA in neurons from male mice (62.77 ± 13.53 AFUs naïve to 77.23 ± 9.51 AFUs day 14, p = 0.4762, n = 3) **(Fig 3E)**. In addition, the area of polarized MHCII per neuron increased >4-fold after PTX in females (5.53% ± 1.26 naïve to 23.67% ± 0.65 day 14, p<0.0001, n = 3) and 2-fold in males (7.34% ± 1.96 naïve to 14.60% ± 0.52 day 14, p = 0.0064, n = 3) **(Fig 3F)**. Collectively, PTX increases surface-MHCII levels in females and polarization of peptide-bound MHCII in female and male mice, suggesting treatment with PTX increases the likelihood that DRG neurons could activate CD4^+^ T cells, particularly in female mice.

### MHCII protein is found in sensory neurons in mouse DRG tissue

**Fig 4:**
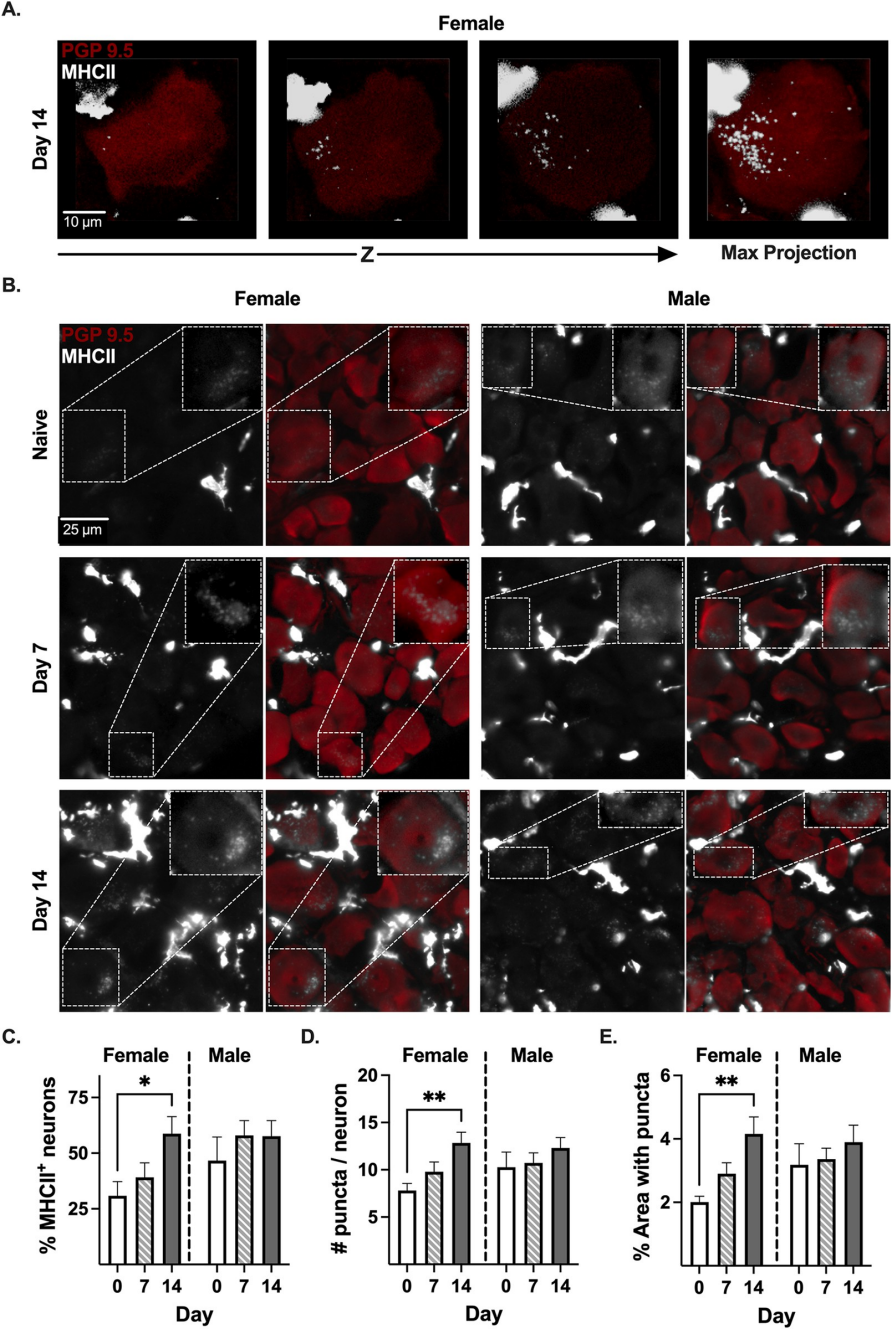
PTX increases neuronal MHCII in female DRG. **(A)** Representative confocal Z-stack and maximum projection IHC images of MHCII (grayscale) in PGP9.5^+^ (red) neuron in L4 DRG tissue from a day 14 PTX-treated female mouse. **(B)** Representative widefield epifluorescence images of DRG tissue from naïve and PTX-treated (day 7 and 14) female and male mice. Inset: representative MHCII^+^ neuron. Quantification of the **(C)** percent of MHCII^+^ neurons, **(D)** number of MHCII puncta/neuron, and **(E)** percent area with MHCII puncta from **(D)**. **(C-E)** Statistical significance determined by 2-way ANOVA with Dunnett’s multiple comparison test (*p <0.05, **p < 0.01, n = 8).

MHCII protein was detected in neurons **(Fig 4A and 4B)** and immune cells **(S5 Fig in S1 File)** in mouse DRG by immunohistochemistry (IHC). Neuronal MHCII was visualized by confocal **(Fig 4A)** and widefield epifluorescence **(Fig 4B)** microscopy. Confocal microscopy demonstrated that the MHCII signal was detected through the entire DRG neuron cell body **(Fig 4A)**, excluding the possibility that the signal was originating from a proximal non-neuronal cell. DRG neurons from both naïve female and male mice expressed MHCII protein (30.84% ± 6.37 in naïve female DRG and 46.64% ± 10.60 in naïve male DRG**) (Fig 4B and 4C)**. While PTX induces the upregulation of MHCII on immune cells [32], the effect on neuronal MHCII is unknown. PTX increased the percent of MHCII^+^ DRG neurons in female mice almost 2-fold (30.84% ± 6.37 in naïve to 58.74% ± 7.69 14 days post-PTX, p = 0.0246, n = 8) **(Fig 4B and 4C)**. In contrast, the percent of MHCII^+^ neurons in male DRG did not increase significantly after administration of PTX (46.64% ± 10.60 in naïve to 57.59% ± 7.01 14 days post-PTX, p = 0.4967, n = 8) **(Fig 4B and 4C)**.

We observed a distinct punctate staining for MHCII in DRG neurons, a pattern consistent with previous reports for immune cells [34]. Therefore, we quantified the number of MHCII puncta in DRG neurons from naïve and PTX-treated female and male mice. There were 7.81 ± 0.75 MHCII puncta/neuron in the DRG from naïve female mice, which significantly increased to 12.84 ± 1.14 MHCII puncta/neuron 14 days post-PTX **(Fig 4D**, p = 0.0063, n = 8**)**. In contrast, the number of MHCII puncta in DRG neurons from male mice remained constant after treatment with PTX (10.28 ± 1.60 in naïve to 12.32 ± 1.1 14 days post-PTX, p = 0.3702, p = 8) **(Fig 4D)**. In addition to the number of MHCII puncta, we measured the percent of neuronal cell area with MHCII puncta. The area with MHCII puncta on DRG neurons increased >2-fold in female mice 14 days post-PTX (2.01% ± 0.18 in naïve to 4.16% ± 0.54 14 days post-PTX, p = 0.0041, n = 8) **(Fig 4E)**. In contrast, the area with MHCII puncta remained constant in DRG neurons from male mice (3.18% ± 0.67 in naïve to 3.90% ± 0.54 14 days post-PTX, p = 0.4510, n = 8) **(Fig 4E)**. Collectively, our data demonstrate that PTX treatment increases the expression of MHCII protein in DRG neurons only in female mice.

### PTX induces the expression of MHCII protein in small diameter DRG neurons in female mice

**Fig 5:**
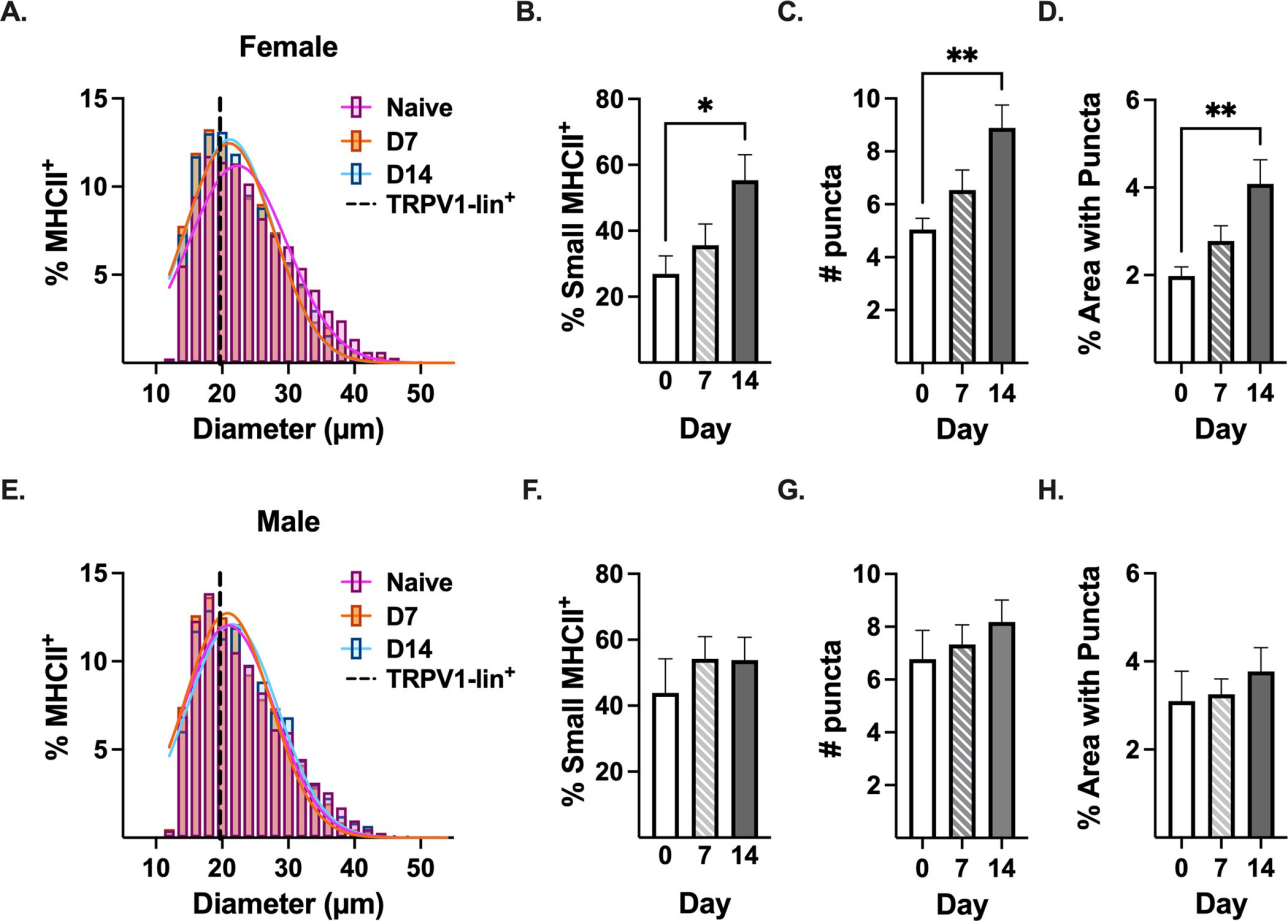
MHCII^+^ DRG neurons are primarily small diameter, and MHCII expression increases in female mice after PTX. **(A, E)** Gaussian distribution of the diameter of MHCII^+^ DRG neurons in DRG tissue from naïve (pink), day 7 (orange) and day 14 (blue) PTX-treated **(A)** female and **(E)** male mice (n = 8, pooled neurons). Dashed black line: diameter mode of TRPV1-lineage td-tomato^+^ neurons **(S6 Fig in S1 File)**. Quantification of **(B, F)** percent of small diameter (≤25 μm) DRG neurons positive for MHCII, **(C, G)** number of MHCII puncta/small diameter neuron, and **(D, H)** percent of small diameter neuron area with MHCII puncta. **(B-D, F-H)** Statistical significance determined by 1-way ANOVA with Dunnett’s multiple comparison test (*p <0.05, **p < 0.01, n = 8).

PTX has been shown to affect both large (Aβ) [35] and small (Aẟ and C) [35] sensory nerve fibers at the peripheral terminals [36] and within the DRG [11, 35]. Although low levels of MHCII transcript have been detected across all neuronal subsets in the DRG [25], it is unknown if the level of MHCII protein is dependent upon the diameter of the neuronal cell body. The diameter of neurons was calculated using an automated analysis pipeline and validated using DRG tissue from TRPV1-lineage td-tomato reporter mice, which identifies TRPV1-lineage neurons as putative nociceptors **(S6 Fig in S1 File)**. TRPV1 is a broad embryonic marker for many small diameter nociceptors including transient receptor potential cation channel subfamily V member (TRPV1), isolectin-B4 (IB4), and a subset of Aẟ neurons [37], so all of these nociceptors will express td-tomato protein even though some subpopulations may lose TRPV1 expression after development (i.e., IB4 and Aẟ) [38]. Although MHCII protein was detected in large diameter neurons (>30μM [39]) in naïve female DRG tissue, the majority of MHCII^+^ neurons were small diameter (<25μM: 63.63% ± 3.056) **(Fig 5A)**. After PTX treatment (7 and 14 days), the histogram of neuron diameter size of MHCII^+^ neurons for female mice shifted left toward the diameter mode of td-tomato^+^ neurons from TRPV1-lineage reporter mice **(Fig 5A)**, indicating that PTX induced MHCII protein in small diameter neurons or reduced MHCII in large diameter neurons. The percent of small diameter neurons that were MHCII^+^ increased >2-fold 14 days after PTX treatment (26.89% ± 5.48 in naïve to 55.37% ± 7.72 14 days post-PTX, p = 0.0117, n = 8**) (Fig 5B)**, while the percent of large diameter neurons that were MHCII^+^ did not significantly increase (43.11% ± 9.23 in naïve to 67.81% ± 7.70 14 days post-PTX, p = .0779, n = 8**) (S7 Fig in S1 File)**, indicating that PTX treatment primarily induces MHCII expression in small diameter neurons in female DRG. Moreover, PTX treatment increased the number of MHCII puncta in small diameter neurons from 5.05 ± 0.42 puncta/neuron in naïve female DRG to 8.89 ± 0.87 puncta/neuron 14 days post-PTX (p = 0.0018, n = 8) **(Fig 5C)** and increased the percent of neuronal area with MHCII puncta >2-fold (1.98% ± 0.21 in naïve to 4.08% ± 0.55 14 days post-PTX, p = 0.0022, n = 8**) (Fig 5D)**, further supporting induction of MHCII in small diameter neurons in female DRG after treatment with PTX.

Like female mice, the majority of MHCII^+^ neurons in naïve male DRG were small diameter (<25μM: 66.00% ± 2.02) **(Fig 5E)**. However, unlike females, the administration of PTX did not change the size distribution of MHCII^+^ DRG neurons (pink naïve line overlaps with orange day 7 and blue day 14 PTX lines) **(Fig 5E)**. Therefore, not surprisingly, administration of PTX in male mice did not significantly change the percent of small (43.86% ± 10.38 in naïve to 53.82% ± 6.90 14 days post-PTX, p = 0.6036, n = 8**) (Fig 5F)** or large (55.27% ± 11.06 in naïve to 69.09% ± 7.41 14 days post-PTX, p = 0.4436, n = 8**) (S7 Fig in S1 File)** diameter neurons that were MHCII^+^. Moreover, PTX treatment in male mice did not change the number of puncta/neuron **(Fig 5G)** or the percent of neuronal area with MHCII puncta **(Fig 5H)**.

### Small diameter nociceptive neurons express MHCII

**Fig 6:**
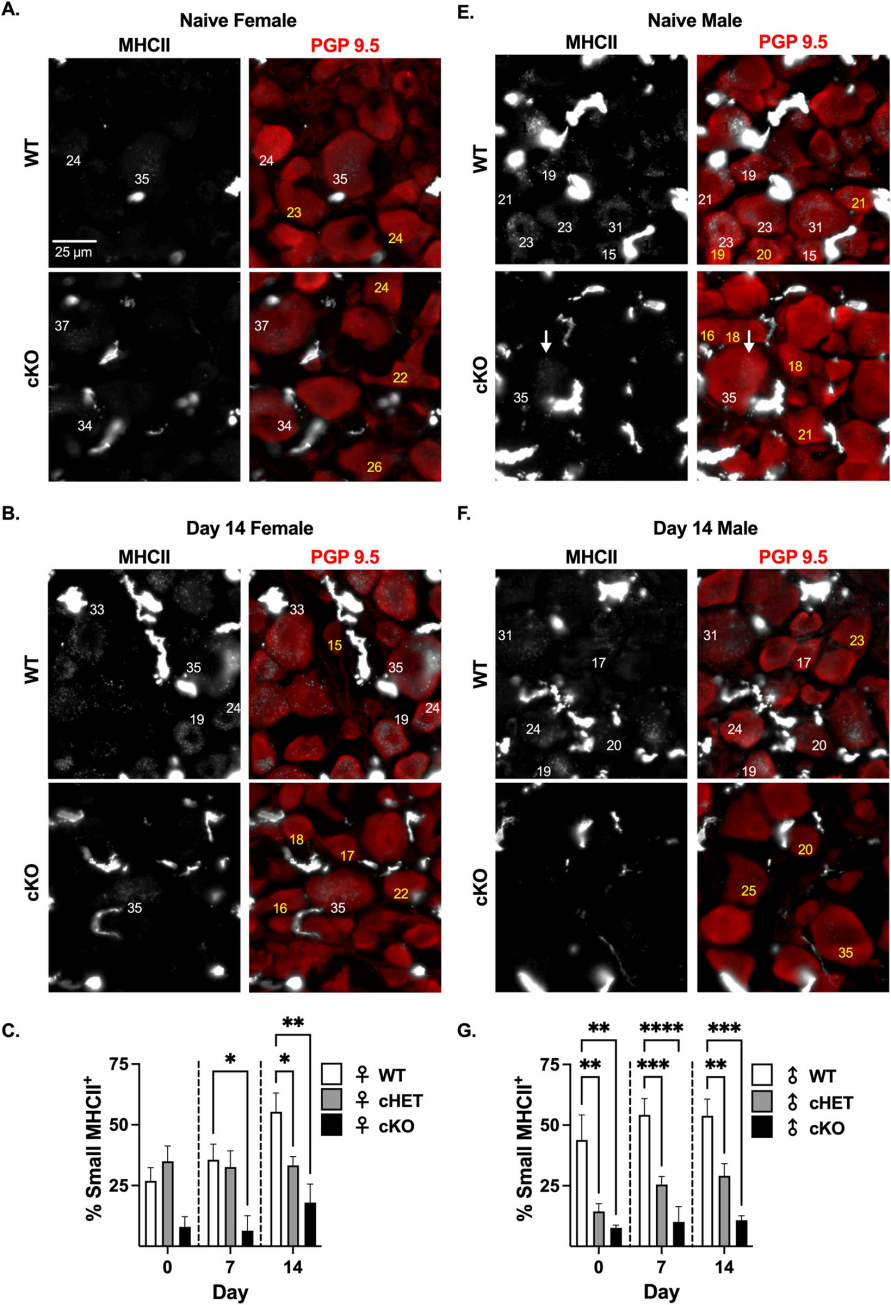
TRPV1-lineage neurons express MHCII in naïve male and female DRG, but PTX treatment only increases MHCII in TRPV1-lineage neurons in female DRG. **(A, B, E, F)** Immunohistochemistry of MHCII (grayscale) and neuronal marker PGP9.5 (red) in L4 DRG tissue from TRPV1-lineage MHCII^+/+^ (WT), MHCII^+/-^ (cHET) and MHCII^-/-^ (cKO) naïve and day 14 PTX-treated female and male mice. Number = neuron diameter size, white (MHCII^+^) and yellow (MHCII^-^). Percent of small diameter MHCII^+^ neurons for **(C)** female and **(G)** male WT, cHET, and cKO mice. **(C, G)** Statistical significance determined by 2-way ANOVA with Dunnett’s multiple comparison test (*p <0.05, **p <0.01, ***p <0.001, ****p <0.0001; n = 8–11 for WT and cHET; n = 4–5 for cKO).

Given the contribution of small nociceptive neurons to CIPN [11], and that the majority of MHCII is expressed in small diameter neurons, we knocked out one copy of MHCII in TRPV1-lineage neurons (putative nociceptors that include TRPV1, IB4, and a subset of Aẟ neurons; **cHET:** TRPV1^lin^ MHCII^+/-^). Knocking out one copy of MHCII in TRPV1-lineage neurons did not change the percent of small diameter MHCII^+^ neurons in the DRG of naïve female mice **(Fig 6C)**, but prevented the PTX-induced increase in small diameter MHCII^+^ neurons in female mice 14 days post-PTX (55.37% ± 7.72 at day 14 in MHCII^+/+^ (wild type: WT) to 33.30% ± 3.61 at day 14 in cHET, p = 0.0231, n = 8) **(Fig 6C)**. Although knocking out one copy of MHCII in TRPV1-lineage neurons in female mice did not significantly reduce the percent of MHCII^+^ small diameter neurons in naïve and 7-days post-PTX mice, knocking out both copies of MHCII (**cKO:** TRPV1^lin^ MHCII^-/-^) drastically reduced the percent of small diameter neurons that were MHCII^+^ by 70% in naïve (26.89% ± 5.48 in WT to 7.95% ± 2.12 in cKO, p = 0.1351, n = 8 WT and n = 4 cKO) and 80% at day 7 (35.58% ± 6.48 in WT to 6.43% ± 3.07 in cKO, p = 0.0135, n = 8 WT and n = 4 cKO) **(Fig 6A and 6C)**. The percent of MHCII^+^ small diameter neurons in cKO female mice remained extremely low 14 days post-PTX (55.37% ± 7.72 at day 14 in WT to 17.99% ± 3.81 at day 14 in cKO, p = 0.0014, n = 8 WT and n = 4 cKO) **(Fig 6B and 6C)**, further supporting that PTX induces MHCII in small diameter nociceptive DRG neurons.

In contrast to female mice, knocking out one copy of MHCII in TRPV1-lineage neurons in male mice reduced the percent of small diameter MHCII^+^ neurons by 66% in naïve mice (43.86% ± 10.38 in WT to 14.38% ± 3.18 in cHET, p = 0.0017, n = 8) **(Fig 6G)**. Like females, knocking out both copies of MHCII in TRPV1-lineage neurons reduced the percent of small diameter MHCII^+^ neurons by 82% in naïve male mice (43.86% ± 10.38 in WT to 7.62% ± 1.16 in cKO, p = 0.0016, n = 8 WT and n = 4 cKO) **(Fig 6E and 6G)**. Knocking out one copy of MHCII^+^ in TRPV1-lineage neurons in male mice reduced the percent of small diameter MHCII^+^ neurons by 53% at day 7 (54.19% ± 6.75 at day 7 in WT to 25.51% ± 3.33 at day 7 in cHET, p = 0.0110, n = 8 WT and n = 11 cHET) and 45% at day 14 (53.82% ± 6.90 at day 14 in WT to 29.09% ± 5.03 at day 14 in cHET, p = 0.0087, n = 8) **(Fig 6G)**. Additionally, knocking out both copies of MHCII in TRPV1-lineage neurons in males reduced the percent of small diameter MHCII^+^ neurons even further, 80% at both 7 days post-PTX (54.19% ± 6.75 at day 7 in WT to 10.04% ± 6.37 at day 7 in cKO, p<0.0001, n = 5 cKO) and 14 days post-PTX (53.82% ± 6.90 at day 14 in WT to 10.77% ± 1.85 at day 14 in cKO, p = 0.0002, n = 4 cKO) **(Fig 6F and 6G)**. By using transgenic mice, we were able to reduce the expression of MHCII in TRPV1-lineage neurons. This finding further supports expression of MHCII protein in DRG nociceptors from both sexes but regulation by PTX in only female mice.

### Reducing MHCII in TRPV1-lineage nociceptors decreases anti-inflammatory CD4^+^ T cells in mouse DRG

**Fig 7:**
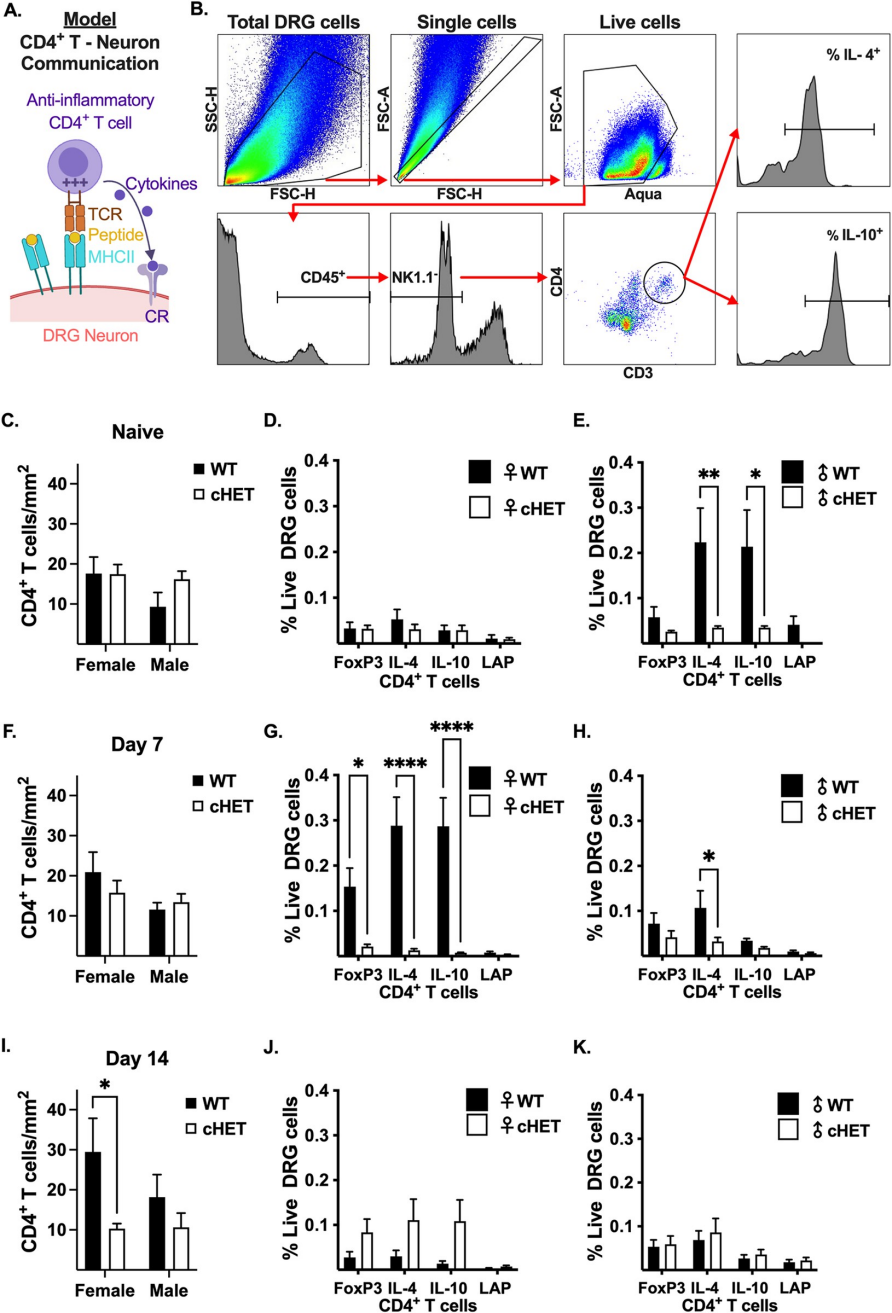
Reducing MHCII in TRPV1-lineage neurons decreases anti-inflammatory CD4^+^ T cells in the DRG of female and male mice. **(A)** Model for CD4^+^ T cell communication with DRG neuron through direct cell-cell contact where TCR binding to neuronal MHCII results in CD4^+^ T cell activation and production of anti-inflammatory cytokines, which act on cytokine receptors (CR) expressed on the neuron. Image created with BioRender.com. **(B)** Nested gating strategy used to identify anti-inflammatory CD45^+^ NK1.1^-^ CD3^+^ CD4^+^ T cells out of total live (Aqua^+^) DRG cells. Quantification of CD4^+^ T cells per mm^2^ of DRG tissue and frequency of anti-inflammatory CD4^+^ T cells out of total live DRG cells for naïve **(C-E)**, 7 days post-PTX **(F-H)**, and 14 days post-PTX **(I-K)** female and male WT and cHET mice. **(C-K)** Statistical significance determined by 2-way ANOVA with Sidak’s multiple comparison test (*p <0.05, **p <0.01, ****p <0.0001; n = 8–9 for CD4^+^ T cell/mm^2^ DRG tissue and n = 6 for % anti-inflammatory CD4^+^ T cells). LAP = latency activated protein.

We hypothesize that neuronal MHCII binds with the T cell receptor (TCR) on CD4^+^ T cells to induce T cell activation, proliferation, and cytokine production **(Fig 7A)**. As a result of this interaction, we would expect mice that lack one copy of MHCII in TRPV1-lineage neurons to have decreased CD4^+^ T cell number and/or decreased cytokine production from CD4^+^ T cells in the DRG. To investigate this hypothesis, we determined the frequency of cytokine producing CD4^+^ T cells in mouse DRG by multi-color flow cytometry **(Fig 7B)** and quantified the number of CD4^+^ T cells per mm^2^ of DRG tissue by IHC **(Fig 7C, 7F and 7I)** for WT and cHET male and female mice. There was no difference in the number of CD4^+^ T cells in the DRG of naïve female WT and cHET mice (17.61 ± 4.13 in WT and 17.44 ± 2.43 in cHET, p = 0.9991, n = 8) or naïve male WT and cHET mice (9.34 ± 3.56 in WT and 16.18 ± 2.04 in cHET male, p = 0.2541, n = 8) **(Fig 7C)**. Likewise, there was no difference in the frequency of anti-inflammatory CD4^+^ T cell subpopulations in the DRG of naïve WT female mice compared to naïve cHET female mice **(Fig 7D)**. In contrast, naïve WT male mice had 6-fold more IL-4 (0.223% ± 0.76 in WT and 0.035% ± 0.004 in cHET, p = 0.0086, n = 6) and IL-10 (0.214% ± 0.081 in WT and 0.035% ± 0.004 in cHET, p = 0.0136, n = 6) producing CD4^+^ T cells in the DRG compared to naïve cHET male mice **(Fig 7E)**. Like naïve mice, there was no difference in the number of CD4^+^ T cells in the DRG of female WT and cHET mice 7 days post-PTX (20.91 ± 4.97 in WT and 15.77 ± 3.05 in cHET, p = 0.4635, n = 8) or male WT and cHET mice 7 days post-PTX (11.56 ± 1.71 in WT and 13.41 ± 2.11 in cHET male, p = 0.8968, n = 8–9) **(Fig 7F)**. However, at 7 days post-PTX, cHET females had a significant decrease in FoxP3^+^ CD4^+^ T cells (>7-fold decrease from 0.153% ± 0.041 in WT to 0.021 ± 0.005 in cHET, p = 0.0407, n = 6), IL-10^+^ CD4^+^ T cells (37-fold decrease from 0.287% ± 0.063 in WT to 0.008 ± 0.001 in cHET, p<0.0001, n = 6) and IL-4^+^ CD4^+^ T cells (22-fold decrease from 0.288% ± 0.063 in WT to 0.013 ± 0.004 in cHET, p<0.0001, n = 6) in the DRG compared to WT female mice **(Fig 7G)**. Similarly, cHET male mice had a significant decrease in IL-4^+^ CD4^+^ T cells in the DRG compared to WT male mice (>3-fold decrease from 0.107% ± 0.038 in WT to 0.032 ± 0.009 in cHET, p = 0.0486, n = 6) **(Fig 7H)**. At 14 days post-PTX, there was no difference in CD4^+^ T cells in the DRG of male WT and cHET mice (18.19 ± 5.62 in WT and 9.35 ± 3.65 in cHET, p = 0.4490, n = 8), but there were 3-fold more CD4^+^ T cells per mm^2^ in the DRG of WT female mice compared to cHET female mice (29.47 ± 8.39 in WT and 10.31 ± 1.25 in cHET, p = 0.0362, n = 8) **(Fig 7I)**. However, there was no difference in the frequency of anti-inflammatory CD4^+^ T cells in the DRG of WT and cHET female **(Fig 7J)** or male **(Fig 7K)** mice. Together, these data support that neuronal MHCII modulates CD4^+^ T cell cytokine production in the DRG of male and female mice.

### MHCII-expressing nociceptors attenuate cold hypersensitivity

Given that small diameter nociceptors contribute to CIPN [11] and cold hypersensitivity is one of the major symptoms [40–42], we determined the extent MHCII expressed in small nociceptive neurons protects against cold hypersensitivity by the thermal placed preference (TPP) behavioral test. Although mice are motivated to explore their environment, mice that are hypersensitive will spend progressively less time on the cold plate with each successive trial as a result of learning. A faster acquisition of a learned avoidance response demonstrates a greater degree of cold hypersensitivity.

**Fig 8:**
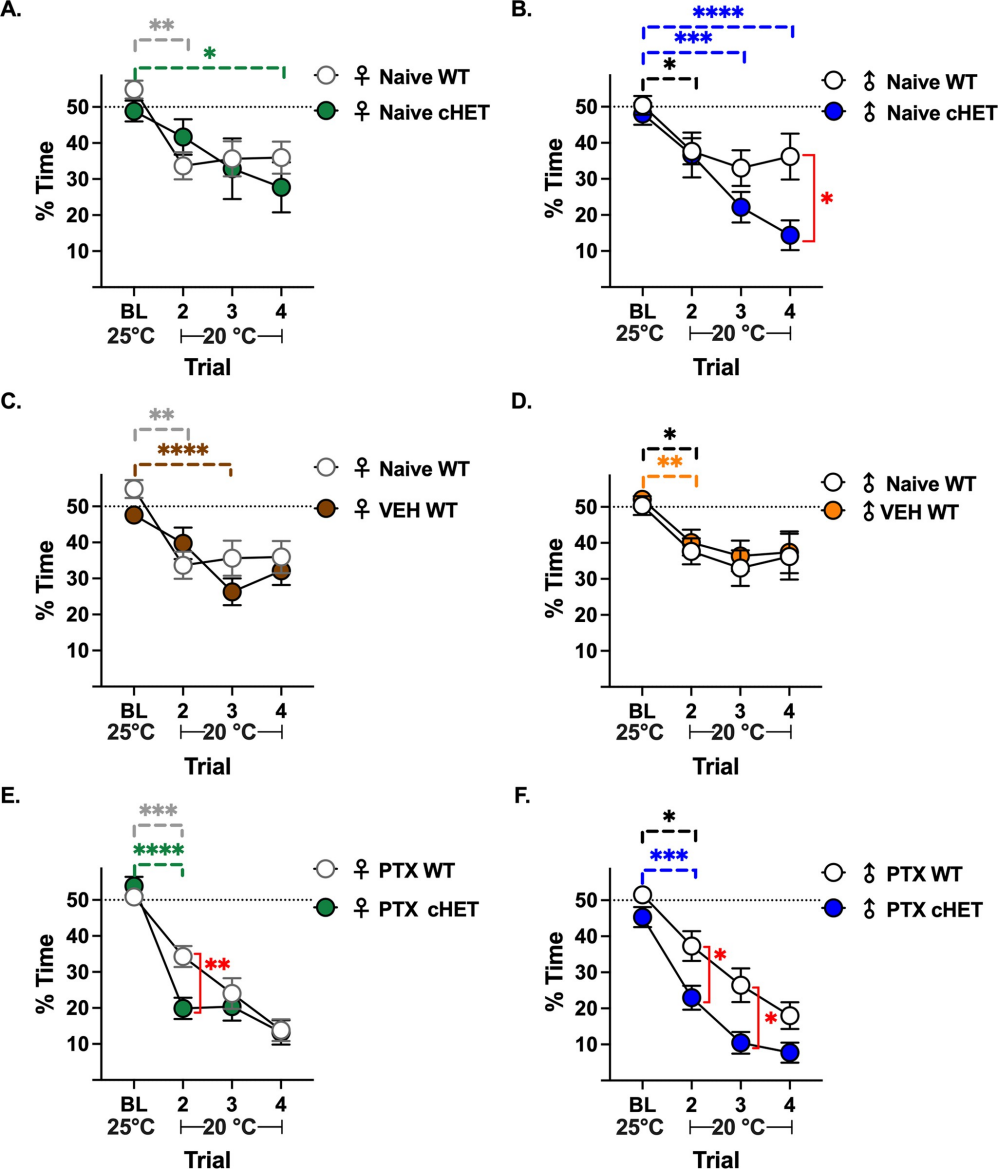
MHCII in TRPV1-lineage neurons attenuates cold hypersensitivity. Thermal placed preference test (test plate = 25°C, baseline (BL) and 20°C, test trials 2–4): Percent of time spent on test plate for naïve **(A, B)**, 6 days post-VEH **(C, D)**, and 6 days post-PTX **(E, F)** WT and cHET female and male mice. Dashed line: expected percent of time on test plate when mice are not hypersensitive to cold. Statistical significance determined by repeated measures mixed-effects model (REML) with Dunnett’s multiple comparison test comparing trials 2–4 to BL within same group (horizontal bars, complete statical analysis in **S1 and S2 Tables in S1 File**), and Sidak’s multiple tests for significance between groups at the same trial (red vertical bars) (*p <0.05, **p <0.01, ***p<0.001, ****p<0.0001 n = 16–19).

Naïve WT female mice were slightly hypersensitive to the cold plate (54.82% ± 2.49 at baseline (BL) to 33.67% ± 3.75 at trial 2, p = 0.0019, n = 17) while knocking out one copy of MHCII in TRPV1-lineage neurons (cHET) induced a slight increase in cold hypersensitivity at trial 4 (48.87% ± 2.86 at BL to 27.71% ± 6.92 at trial 4, p = 0.0127, n = 16) **(Fig 8A)**. In contrast, reducing one copy of MHCII in TRPV1-lineage neurons (cHET) in naïve male mice drastically increased cold hypersensitivity compared to naïve WT male mice (50.36% ± 2.60 at BL to 37.66% ± 3.63 at trial 2, p = 0.0198, n = 19) **(Fig 8B)**. With each successive trial, the TRPV1-lineage cHET male mice spent less time on the cold plate with a significant difference between BL (48.03% ± 3.02) and trial 3 (22.15% ± 4.24, p = 0.0005, n = 16) but were not significantly different from WT mice until trial 4 (36.20% ± 6.38 for WT and 14.40% ± 4.13 for cHET, p = 0.0296, n = 16–19) **(Fig 8B)**. This enhanced learning pattern to avoid the cold plate in cHET male mice indicates cold hypersensitivity, demonstrating that expression of MHCII in small nociceptive neurons prevents cold hypersensitivity in naïve male mice.

Next, we sought to determine the extent PTX exacerbates cold hypersensitivity in WT and cHET male and female mice; however, first we had to confirm that the vehicle (VEH = cremophor:ethanol) that was used to dissolve PTX did not induce cold hypersensitivity on its own. There were no differences in cold hypersensitivity between naïve female **(Fig 8C)** and male **(Fig 8D)** mice and VEH-treated mice (6 days post-VEH injection). Administration of PTX induced cold hypersensitivity in WT (50.83% ± 1.62 at BL to 34.30% ± 2.91 at trial 2, p = 0.0003, n = 16) and cHET (53.87% ± 2.55 at BL to 19.88% ± 2.97 at trial 2, p<0.0001, n = 16) female mice by trial 2; however cHET female mice were statistically more hypersensitive than WT female mice (34.30% ± 2.91 for WT and 19.88% ± 2.97 for cHET, p<0.0001, n = 16) **(Fig 8E)**. Like female mice, administration of PTX induced cold hypersensitivity in WT (51.50 ± 1.44 at BL to 37. 30 ± 4.13, p = 0.0167, n = 16) and cHET (45.31 ± 2.78 at BL to 22.96 ± 3.34 at trial 2 in cHET, p = 0.0005, n = 16) male mice at trial 2 **(Fig 8F)**; however, cHET male mice were more hypersensitive to cold temperature compared to WT male mice at both trial 2 (37.30% ± 4.13 for WT and 22.96% ± 3.34 for cHET, p = 0.0453, n = 16) and trial 3 (26.43% ± 4.67 for WT and 10.44% ± 2.99 for cHET, p = 0.0311, n = 16) **(Fig 8D)**. Expression of MHCII in small diameter TRPV1-lineage nociceptors (TRPV1, IB4, and a subset of Aẟ) attenuates PTX-induced cold hypersensitivity in both female and male mice.

## Discussion

The debilitating nature of CIPN has been a major obstacle for the continuation of life-saving measures for individuals with cancer. Recently, it has been shown that anti-inflammatory cytokines (IL-10 and IL-4) are protective against CIPN [15, 43]; however, systemic use of IL-10 in general has had poor clinical outcomes [44] as it can promote the progression of cancer [45] and propagate chronic viral infections [46]. Therefore, it would be beneficial to develop therapies that promote endogenous IL-10 and IL-4 production locally in the nervous system. In our recent work, we found that PTX induces a robust increase in anti-inflammatory (IL-10^+^ and IL-4^+^) CD4^+^ T cells in the DRG of female mice [18], but the mechanism by which CD4^+^ T cells are activated and the extent cytokines released by CD4^+^ T cells target neurons in the DRG are unknown. We hypothesize that PTX exposure increases MHCII on sensory neurons, which would stimulate the paracrine release of anti-nociceptive cytokines by CD4^+^ T cells to suppress CIPN **(Fig 7A, Model)**.

MHCII is traditionally thought to be constitutively expressed only in APCs but can be induced by inflammation in non-APCs [27]. RNA seq data sets [23–25, 31] demonstrate that mouse and human DRG neurons express transcripts for MHCII and MHCII-associated genes. However, there are no reports to date that demonstrate MHCII protein expression in terminally differentiated neurons. For the first time, we show expression of functional MHCII protein in mouse DRG neurons. Neuronal MHCII was most likely overlooked in the P. Hu et al. study as MHCII^+^ cells in the DRG were identified based on morphology instead of co-staining with cell-specific markers. Additionally, the signal intensity of immune cell MHCII is >5 times than neuronal MHCII, indicating a lower density of MHCII molecules in neurons. Therefore, if not actively investigating neuronal MHCII, the settings used to capture immune cell MHCII would obscure neuronal MHCII. In addition, trying to identify a few molecules of MHCII is technically challenging and a limitation of IHC as the signal is not much higher than background. Thus, it is likely that neurons with extremely low levels of MHCII were classified as negative cells in our study. Even though DRG neurons have fewer MHCII molecules per cell area than non-neuronal cells, it is likely still functionally significant given that clustering allows for as little as 210 MHCII molecules to activate a CD4^+^ T cell [47, 48]. Expression of MHCII in neurons will provide insight into the mechanism by which CD4^+^ T cells contribute to pain, autoimmunity, and neurological diseases.

We found that MHCII is primarily expressed in small diameter neurons; however, the regulation of MHCII expression differs between male and female mice. To investigate the extent MHCII was expressed in nociceptors, we knocked out MHCII in TRPV1-lineage neurons. Knocking out one copy of MHCII in TRPV1-lineage neurons in naive male mice decreased the percent of MHCII^+^ DRG neurons. In contrast, knocking out one copy of MHCII in TRPV1-lineage neurons in female mice did not change the percent of MHCII^+^ DRG neurons. As MHCII molecules are codominantly expressed, we expected cHET female mice to have reduced MHCII^+^ neurons as we saw for cHET male mice. When this did not occur, we concluded that neuronal MHCII^+^ must be expressed in non-TRPV-1 lineage small diameter neurons in naïve female mice. However, by knocking out both copies of MHCII in TRPV1-lineage neurons, the percent of MHCII^+^ neurons in the DRG of naïve female mice was severely reduced (70%), suggesting MHCII was indeed expressed in TRPV1-lineage neurons but only one parental allele was being expressed. Processes including X chromosome inactivation, random monoallelic expression, or allelic exclusion are known to cause this allelic imbalance or allele-specific expression (ASE) [49]. Epigenetic modifications (DNA methylation, histone modification, and microRNAs) contribute to ASE, and inflammatory mediators, specifically IFN-γ, are known to promote chromatin remodeling to enhance MHC expression [50]. Thus, we predict that the heightened inflammatory response at day 14 promoted expression of MHCII by both parental alleles in female WT mice and therefore, the percent MHCII^+^ DRG neurons in day 14 PTX-treated cHET female mice was reduced by half. Future studies will investigate sex-specific epigenetic regulation of MHCII in neurons and how it changes under inflammatory conditions.

In addition to cytokines, engagement of TLRs can increase MHCII expression [51, 52]. While DRG neurons from both male and female mice express transcripts and/or protein for TLRs 1–9 [31, 53–57], female mice have >5-fold more TLR4-6 transcripts than male mice [31]. Thomas A Szabo-Pardi et al demonstrate that TLR4 signaling in DRG neurons mediates mechanical hypersensitivity after a nerve injury in only female mice [58], further supporting a sex-specific role for TLR4 in the DRG. Here, we report a sex-specific difference in the regulation of neuronal MHCII, though the mechanism by which PTX induces MHCII in small diameter neurons in only female mice remains unknown. TLR4 binds to PTX [6], and TLR4 engagement increases surface-MHCII in APCs [51], together suggesting one potential mechanism by which PTX treatment increases MHCII in DRG neurons in female mice.

Differential MHCII expression contributes to cellular function [59]. For example, low MHCII levels have been shown to promote anti-inflammatory CD4^+^ T cell responses [59], which we see in this study with DRG neurons. While the frequencies of anti-inflammatory CD4^+^ T cells within the CD4^+^ T cells gate for male and female naïve mice were consistent with our previous publication [18], the frequencies of anti-inflammatory CD4^+^ T cells out of total live DRG cells in this study were lower in naïve females and higher in naïve males [18]. Although not significant, we now show that naïve WT female mice tend to have more CD4^+^ T cells per mm^2^ in DRG tissue compared to WT naïve male mice **(Fig 7C)**, suggesting that female mice in this cohort could have more non-CD4^+^ T immune cells in the DRG and male mice have fewer. Collectively, these data indicate that multiple anti-inflammatory immune cell populations may contribute to the maintenance of homeostasis in the DRG.

Chemokines attract different immune populations to the DRG, and DRG neurons express RNA transcripts for chemokines CCL19/21/27, which are potent chemoattractants for CD4^+^ T cells. Recently, CCL19/21 have been shown to induce the migration of CD4^+^ T cells to non-lymphoid tissue, especially to the nervous system [60, 61], to preserve the balance between immune surveillance (against tumor and pathogens) and tolerance. The presence of CD4^+^ T cells in naïve mouse DRG [18, 62], together with the expression of MHCII in neurons support direct cell-cell interaction, despite SGCs surrounding DRG neurons [29]. Our present work demonstrates that CD4^+^ T cells can breach the SGC barrier, most likely through a natural gap in the surrounding glial envelope or a gap junction between adjacent glial cells. CD4^+^ T cells in close proximity to DRG neurons in the naïve mouse suggests a role for CD4^+^ T cells in not only immune surveillance and tolerance, but also regulating neuronal function. Neuroimmune communication likely occurs in pathological conditions as nerve injury and nerve transection models demonstrate that macrophages can breach SGCs and lie directly against the neuron in the DRG [30] and trigeminal ganglion [63]. Similarly, we found CD4^+^ T cells breached the SGCs barrier after treatment with PTX. We hypothesize that neuronal MHCII-dependent CD4^+^ T cell activation would target cytokine release toward neurons to suppress hypersensitivity after inflammation and/or nerve injury.

Neuronal MHCII is primarily expressed in small diameter nociceptors, which have been shown to contribute to CIPN [11]. As cold hypersensitivity is a major symptom of CIPN [40–42], we assessed the contribution of MHCII in small diameter nociceptive neurons in naïve and PTX treated mice by TPP. TPP is a behavioral test that incorporates the ability to choose and the potential to learn, demonstrating higher order pain processing. Although naïve male mice tend to have more MHCII^+^ neurons in the DRG than naïve female mice, female mice tend to have more CD4^+^ T cells per mm^2^ of DRG tissue. The increased number of CD4^+^ T cells may in part explain why we did not see a difference in cold sensitivity between naïve female and male mice. Additionally, other anti-inflammatory immune populations may prevent cold hypersensitivity as previous reports have shown that CD8^+^ T cells and IL-10 producing macrophages in the DRG suppress CIPN [64].

CIPN is exacerbated in IL-10 [15] and IL-4 [43] knockout mice, emphasizing a critical role for IL-10 and IL-4 in suppressing CIPN. Moreover, application of IL-10 to cultured DRG neurons suppressed PTX-induced spontaneous discharges [15], demonstrating a direct effect of anti-inflammatory cytokines on neuronal function. After PTX administration, the frequency of anti-inflammatory CD4^+^ T cells (IL-10 and IL-4) in the DRG was significantly less in male and female mice that lacked one copy of MHCII in TRPV1-lineage neurons compared to WT mice. Furthermore, PTX increased the frequency of anti-inflammatory CD4^+^ T cells in the DRG of WT female mice (consistent with our previous publication [18]), which was prevented by knocking out one copy of MHCII in TRPV1-lineage neurons. Moreover, WT female mice had significantly more CD4^+^ T cells in the DRG 14 days post-PTX than cHET female mice, implicating a potential role for CD4^+^ T cells in the resolution of CIPN and/or susceptibility to future neuropathy. Overall, cHET male and female mice, which had fewer anti-inflammatory IL-4 and IL-10 producing CD4^+^ T cells in the DRG, had more severe PTX-induced cold hypersensitivity. Future studies will evaluate the role of MHCII in PTX-induced mechanical hypersensitivity, another prominent feature of CIPN.

In addition to TRPV1-lineage neurons, we found that both female and male mice express MHCII in large diameter neurons, which primarily convey non-nociceptive input (proprioception, touch, pressure, vibration) from the periphery to the spinal cord but may contribute to nociceptive circuits following tissue or nerve injury as in CIPN [35]. MHCII expression in large diameter neurons indicate that PTX-induced cold hypersensitivity may be even greater if MHCII is eliminated from all neurons and suggests a role in proprioception, pressure, and touch (light, gentle and affective). Future studies will address the role of MHCII in large and small diameter neurons in pain and other sensory modalities.

Neuronal MHCII represents a novel mechanism to suppress CIPN, which could be exploited for therapeutic intervention against not only pain but autoimmunity and neurological diseases. Moreover, future investigation will evaluate the extent direct cell-contact between neuronal MHCII and the TCR facilitates CD4^+^ T cell activation and cytokine production during homeostasis and inflammation/nerve injury.

## Conclusion

Expression of functional MHCII protein was detected on the surface of DRG neurons, suggesting a potential mechanism for CD4^+^ T cell activation and targeted cytokine release. Reducing MHCII from a subpopulation of neurons known to contribute to CIPN decreased anti-inflammatory CD4^+^ T cells in the DRG and increased the severity of PTX-induced cold hypersensitivity in female and male mice.

## Materials and methods

### Mice

Male and female mice were purchased from Jackson Laboratory **(S3 Table in S1 File)**. Male TRPV1^Cre^ mice were bred to female MHCII^fl/fl^ mice to generate TRPV1^lin^ MHCII^+/-^ heterozygote mice **(cHET)**. cHET female mice were mated back to MHCII^fl/fl^ male mice to delete MHCII from TPRV1-lineage neurons, which identifies putative nociceptors that include IB4, TRPV1, and a subset of Aẟ neurons (TRPV1^lin^ MHCII^-/-^ mice; **cKO**). To confirm the presence of MHCII^fl/fl^ and Cre, mouse tails were digested with 50mM NaOH/50mM HCl/1M Tris-HCl, pH 8.0. Primers (**S4 Table in S1 File**) and Promega GoTaq® G2 Hot Start Polymerase were used in PCR to detect MHCII^fl/fl^ (199 base pair), MHCII^fl/+^ (199 and 295 base pairs), and wild type MHCII^+/+^ (295 base pair) mice. PCR using GoTaq® Polymerase determined the presence of Cre **(S3 Table in S1 File)**. cHET×MHCII^fl/fl^ crosses only yielded 12% cKO mice (6% per sex) instead of the predicted 25% (12.5% per sex) based on normal Mendelian genetics. Thus, cKO mice were only used to validate MHCII protein in small nociceptive neurons.

All mice were given food and water *ad libitum*. All experimental protocols followed National Institutes of Health guidelines and were approved by the University of New England Institutional Animal Use and Care Committee. Mice were euthanized with an overdose of avertin (0.5cc of 20 mg/ml) followed by transcardiac perfusion with 1× phosphate buffered saline (PBS). This method of euthanasia is consistent with American Veterinary Medical Association Guidelines for the Euthanasia of Animals.

### Paclitaxel and Thermal Place Preference test (TPP)

PTX (Sigma-Aldrich) was solubilized in cremophor:ethanol 1:1 and diluted 1/3 in 1× PBS (VEH). A single injection of 6mg/kg PTX or VEH at a volume of 10ml/kg bodyweight was given on day 0 as done previously [18, 62]. Mice were randomly assigned a treatment, which experimenters were blinded to during testing. The TPP test was used to quantify non-evoked measures of cold hypersensitivity before and after PTX. Mice were habituated to the room and testing apparatus, which includes two adjustable thermal plates (test and reference). Mice were placed on the reference plate to begin each 3-minute trial. The reference plate side was the same for all trials for an individual mouse, but the sides for the reference and test plates were counterbalanced within each group to exclude a potential side preference. For the habituation trial (#1), both the test and reference plates were set to 25°C, and the mice were allowed to explore for 3 minutes. Any mouse that spent <30 seconds on the test plate during the habituation trial was excluded from further testing. For trials #2–4, the reference plate was set to 25°C and the test plate to 20°C. The percent of time the mice spent on the test plate was reported for each trial. Separate cohorts of mice were run for naive and 6 days post-PTX/VEH to prevent confounding effects of avoidance learning during the naive session impacting the day 6 results.

### Immunohistochemistry (IHC)

Mice were perfused with 1× PBS followed by 4% paraformaldehyde (PFA; Sigma-Aldrich). L4 DRGs were post-fixed in 4% PFA for 1–2 hours, then transferred to 30% sucrose/0.02% sodium azide at 4°C. DRGs were embedded in Clear Frozen Selection Compound prior to serially sectioning into 12 μm slices. DRGs were permeabilized with 1× PBS/0.1% Triton X-100 (PBS-T; Sigma-Aldrich) for 15 minutes at room temperature (RT) and incubated with blocking buffer (PBS-T plus 5% normal donkey serum) for 1 hour at RT. Slides were incubated with primary antibodies **(S5 Table in S1 File)** in blocking buffer overnight at RT in a humidified, light-protected chamber. Slides were washed 3X with blocking buffer and secondary antibodies **(S5 Table in S1 File)** added for 1 hour at RT. Slides were washed 3X with 1× PBS, then cover slipped with Fluoroshield Mounting Medium (±DAPI, Abcam). RFX1 images were acquired with a 20× objective (HC PL Fluotar NA 0.40) on an ImageXpress Pico (Molecular Devices) automated widefield fluorescence microscope with a high-sensitivity, 5-megapixel CMOS camera and using CellReporterXpress Software (CRX; version 2.9.3). Widefield CD4^+^ T cell frequency images were acquired with a 20× (HC PL Fluotar NA 0.40; Z-stacks with 2 μm step) or 40× objective (HC PL Fluotar NA 0.60 with correction ring; Z-stacks with 1 μm step; **Fig 1A**) on an ImageXpress Pico and processed by Best Focus projections in CRX prior to export. MHCII images were acquired with a 40× objective (Plan Fluor NA 0.75) on a Keyence BZ-X710 automated widefield fluorescence microscope with incorporated cooled monochrome CCD camera (960×720 image resolution, 14-bit gradation). Confocal images of CD4^+^ T cell-DRG neurons (IHC), neuronal MHCII ICC, and neuronal MHCII IHC were acquired with a Leica TCS SP5 II confocal microscope using a 40× objective (HCX PL APO NA 1.3 under oil, RI 1.52, zoom 3.0, 0.34 μm step size, 12-bit, scan format either 512×512 or 1024×512). 3D volume of CD4^+^ T cell and DRG neuron was generated by MetaXpress software (Molecular Devices, version 6.7.1.157) using quadratic blending for channel display.

### IHC automated image analysis

IHC analysis was performed using an automated pipeline to eliminate human bias and validated manually by two independent blinded experimenters.

#### CD4^+^ T cells per mm^2^ DRG tissue

Images were imported into MetaXpress analysis software, and a Custom Module was designed to quantify the number of CD4^+^ T cells in DRG tissue (per mm^2^). CD4^+^ T cells were identified as follows: 1) A mask was generated to identify neuron containing regions of tissue. 2) Separate masks were created to identify CD3^+^ and CD4^+^ cells by size and signal intensity. 3) Cells that were positive for CD3 and CD4 were identified as CD4^+^ T cells. 4) Automated image analysis measured the neuron-containing tissue area (mm^2^) and counted CD4^+^ T cells within neuron-containing tissue. Total CD4^+^ T cells were divided by neuron-containing tissue area to measure CD4^+^ T cells per mm^2^ for each mouse (7.119 ± 0.2102 DRG sections/mouse).

#### MHCII^+^ DRG neurons

Keyence images were imported into Fiji [65] to generate single channel images, then analyzed by MetaXpress software. The percent of MHCII^+^ DRG neurons was quantified by developing a Custom Module within MetaXpress. MHCII^+^ DRG neurons were identified as follows: 1) A mask was generated to capture neurons within the DRG tissue while excluding nerve fibers. 2) A subsequent mask was created to identify single neurons based on size and PGP9.5 intensity. 3) A “Shrink” function, which decreased the borders of the neuron mask by 4 pixels, was applied to exclude potential MHCII signal from surrounding SGCs. 4) A mask to capture MHCII^+^ non-neuronal cells was created based on size and signal intensity, which was >5 times greater than neuronal MHCII (≥25,000 arbitrary fluorescent units (AFUs)). 5) The “Grow” function, which expanded the borders of the non-neuronal mask by 7 pixels, was applied to capture low-intensity non-neuronal MHCII staining. 6) To exclude non-neuronal MHCII signal that overlapped DRG neurons, a logical operation was applied. Neurons excluding non-neuronal cell overlap were used to calculate MPI of MHCII in each neuron. 7) The threshold for MHCII^+^ DRG neurons was set as the mean intensity for the MHCII signal at the 99^th^ percentile for DRG L4 stained with an isotype control antibody (n = 3 naïve females). Automated analysis was used to measure the expression of MHCII across all conditions (872.1 ± 25.28 neurons/mouse).

#### MHCII puncta and neuron diameter

MHCII puncta were identified within the neuronal mask as regions of ≥1.5 pixels containing MHCII signal ≥800 MPI above local background. The number of MHCII puncta was determined by measuring the number of puncta per neuron **(Figs 4D, 5C and 5G).** The percent of neuronal area with MHCII puncta was calculated by dividing the sum of puncta-containing area (pixel^2^) by the total area of the neuron **(Figs 4E, 5D and 5H)**. The diameter of DRG neurons was determined by: (1) Measuring the total area of each neuron (pixel^2^); (2) Multiplying the area (pixel^2^) by 6.718 μm^2^/pixel^2^ to convert to μm^2^; (3) using the equation (A = πr^2^).

#### RFX1^+^ DRG neuron

Images were imported into MetaXpress analysis software for automated analysis using a Custom Module as follows: 1) As described for the neuronal MHCII^+^ automated analysis, masks were created to identify DRG tissue and neurons. 2) Separate masks for non-neuronal and neuronal nuclei were created based on nuclei size and intensity of DAPI. 3) Non-neuronal nuclei were removed from the neuron mask using a logical operation. 4) The number of PGP9.5^+^ neurons with a visible nucleus that had nuclear RFX1 with a MPI >37.19 were counted and divided by the total number of neurons with visible nuclei to determine the percent of RFX1^+^ DRG neurons.

### Immunocytochemistr (ICC)

DRGs collected from naïve and day 14 PTX-treated male and female mice were pooled for each mouse and acutely dissociated into a single cell suspension as described previously [18]. DRG neurons were isolated by depleting non-neuronal cells through MACS following the manufacturer’s protocol (Miltenyi). MACS-DRG neurons were resuspended in F12 media supplemented with 10% fetal bovine serum (FBS) and 100 μg/ml primocin (Invivogen) and plated on chamber slides (Millipore) coated with 20 μg/ml laminin (Sigma) and 50 μg/ml poly-D-lysine (Thermo).

#### Total MHCII

After an 18-hour incubation at 37°C, MACS-neurons were washed with 1× PBS and fixed with 4% PFA. Cells were washed with 1× PBS and incubated with blocking buffer for 30 minutes at RT. PGP9.5 was added to the cells for 1 hour at RT. Cells were washed with 1× PBS and incubated with anti-rabbit 488 secondary antibody and MHCII for 1 hour at RT. Slides were imaged with a Leica TCS SP5 II confocal microscope using a 40× objective under oil (HCX PL APO NA 1.3 under oil, RI 1.52, xy resolution 0.199 μm, z resolution 0.814 μm, zoom 3.0, 0.34 μm step size, 12-bit, scan format 1024×512).

#### Extracellular MHCII and OVA peptide

After an 18-hour incubation at 37°C, media was replaced with F12/10% FBS/100 μg/ml primocin. FITC-conjugated OVA peptide (Anaspec) was added at 10 μg/ml for 30 minutes at 37°C. Cells were washed with 1× PBS/1% FBS and blocked with CD16/32 (Biolegend). After 10 minutes, cells were incubated with MHCII for 30 minutes on ice to stain for extracellular MHCII. Cells were washed, fixed with 4% PFA, and stained with PGP9.5. Slides were imaged on the Keyence microscope with a 20× objective.

#### ICC automated image analysis

MHCII on the surface of cultured DRG neurons was quantified using a Custom Module developed in the MetaXpress analysis software. DRG neurons were identified using a mask based on cell size and PGP9.5 intensity. Then the MPI for MHCII and OVA were measured for each neuron. MHCII polarization was quantified as the accumulation of surface MHCII molecules within a defined area (≥ 2 pixels) with a signal intensity ≥ 1.7-fold over local background. Polarized MHCII area was divided by the total DRG neuron area to give the percent neuron area containing polarized MHCII.

### Flow cytometry

DRG cells from naïve, day 7, and day 14 PTX-treated female and male WT and cHET mice were incubated at RT for 30 min with Live/Dead Fixable Violet (Thermo). Cells were pre-incubated with CD16/32 for 10 min prior to the addition of antibodies or isotype **(S5 Table in S1 File)** for 20 min at 4°C. Cells were washed with FACs buffer (1× PBS, 1% FBS) and fixed at RT for 20 min with Intracellular Staining Fixation Buffer (BioLegend). Cells were washed with 2× Cyto-FastTM Perm Wash solution (CFPWS; BioLegend) and incubated with PGP9.5 for 20 min at RT. Cells were washed with 2× CFPWS and incubated with donkey anti-rabbit Cy3 for 20 min at RT. VersaComp Antibody Capture Kit (Beckman Coulter) was used to set compensation to correct for spectral overlap. Unstained DRG cells were used to check for auto-fluorescence. Beckman Coulter Cyto-FLEX S System B2-R3-V4-Y4 was used to acquire >100,000 events in the live (Aqua^+^) DRG cells. Data was analyzed with FlowJo^TM^ 10.8.2.

### Western blot

MACS-DRG neurons from naïve and day 14 PTX-treated female and male mice were lysed in DiGE lysis buffer (7 M urea, 2 M thiourea, 4% CHAPS, 30 mM Tris-HCl) supplemented with 1× Halt Protease and Phosphatase Inhibitor Cocktail (Thermo). Lysates were quantified with the EZQ Protein Quantification Kit (Thermo). Lysates (20 μg) were added to Bolt bis-Tris 12% gel (Thermo). SDS-PAGE was performed using 1× MOPS SDS Running Buffer (Life Technologies). Proteins were transferred to Immobilon-FL PVDF membrane (Millipore) using 1× Power Blotter 1-Step Transfer Buffer (Invitrogen). PVDF membranes were incubated at 4°C for 48 hours with MHCII and beta tubulin as the loading control, then incubated with 1:2000 donkey anti-rabbit Cy5 secondary antibody for 2 hours at RT. PVDF membranes were imaged using the Typhoon 9600 laser scanner (GE). Band intensities were quantified using AutoQuant imaging software.

### Statistical analysis

All experiments were analyzed using Graphpad Prism 9 (Graphpad Software, Inc) and reported as mean ± standard error of mean. A two-way ANOVA with Dunnett’s multiple comparison test was used to compare the means of the number of CD4^+^ T cells per mm^2^ of DRG tissue **(Fig 1)**. A two-way ANOVA with Sidak’s multiple comparison test was used to compare the means of neuronal MHCII from naïve and day 14 PTX-treated female and male mice for western blot, ICC, and flow cytometry (**Figs 2 and 3**) as well as to compare the means between WT and cHET female and male mice for IHC and flow cytometry **(Fig 7)**. An unpaired t-test was used to compare the means of neuronal RFX1 for male and female mice (**S3 Fig in S1 File**). A two-way ANOVA with Dunnett’s multiple comparison test was used to compare the means of MHCII (%, # of puncta, area of puncta) for PTX-treated (day 7 and 14) male and female mice compared to the naïve control (**Figs 4 and 6**). A one-way ANOVA with Dunnett’s multiple comparison test was used within each sex to compare the mean of the percent of small/large diameter MHCII^+^ DRG neurons from PTX-treated mice (day 7 and 14) to the naïve control (**Fig 5 and S7 Fig in S1 File**). TPP behavior was analyzed using a repeated measures mixed-effects model (REML) with Dunnett’s multiple comparison test comparing the mean percent of time on the cold plate (trials #2–4) to baseline (#1) within the same group. Sidak’s multiple comparison test was used to compare the means between two groups within the same trial **(Fig 8)**.

## Supporting information

S1 File (PDF)

S1 Video CD4^+^ T cell breaches the SGC barrier in female mouse DRG tissue. (MP4)

S1 Raw images Original images for western blots. (PDF)

S1 Data All raw data values shown in this study. (XLSX)

S1 Graphical abstract Created with Biorender.com. (TIFF)

## References

[1] Weaver BA. How Taxol/paclitaxel kills cancer cells. Mol Biol Cell. 2014;25(18):2677–81. doi: 10.1091/mbc.E14-04-0916 25213191 PMC4161504

[2] Seretny M, Currie GL, Sena ES, Ramnarine S, Grant R, MacLeod MR, et al. Incidence, prevalence, and predictors of chemotherapy-induced peripheral neuropathy: A systematic review and meta-analysis. Pain. 2014;155(12):2461–70. doi: 10.1016/j.pain.2014.09.020 25261162

[3] Dougherty PM, Cata JP, Cordella JV, Burton A, Weng HR. Taxol-induced sensory disturbance is characterized by preferential impairment of myelinated fiber function in cancer patients. Pain. 2004;109(1–2):132–42. doi: 10.1016/j.pain.2004.01.021 15082135

[4] Jimenez-Andrade JM, Herrera MB, Ghilardi JR, Vardanyan M, Melemedjian OK, Mantyh PW. Vascularization of the dorsal root ganglia and peripheral nerve of the mouse: implications for chemical-induced peripheral sensory neuropathies. Mol Pain. 2008;4:10. doi: 10.1186/1744-8069-4-10 18353190 PMC2289805

[5] Hirakawa H, Okajima S, Nagaoka T, Kubo T, Takamatsu T, Oyamada M. Regional differences in blood-nerve barrier function and tight-junction protein expression within the rat dorsal root ganglion. Neuroreport. 2004;15(3):405–8. doi: 10.1097/00001756-200403010-00004 15094492

[6] Byrd-Leifer CA, Block EF, Takeda K, Akira S, Ding A. The role of MyD88 and TLR4 in the LPS-mimetic activity of Taxol. Eur J Immunol. 2001;31(8):2448–57. doi: 10.1002/1521-4141(200108)31:8&lt;2448::aid-immu2448&gt;3.0.co;2-n 11500829

[7] Zhang H, Li Y, de Carvalho-Barbosa M, Kavelaars A, Heijnen CJ, Albrecht PJ, et al. Dorsal Root Ganglion Infiltration by Macrophages Contributes to Paclitaxel Chemotherapy-Induced Peripheral Neuropathy. J Pain. 2016;17(7):775–86. 26979998 10.1016/j.jpain.2016.02.011 PMC4939513

[8] Kalynovska N, Diallo M, Sotakova-Kasparova D, Palecek J. Losartan attenuates neuroinflammation and neuropathic pain in paclitaxel-induced peripheral neuropathy. J Cell Mol Med. 2020;24(14):7949–58. doi: 10.1111/jcmm.15427 32485058 PMC7348151

[9] Li Y, North RY, Rhines LD, Tatsui CE, Rao G, Edwards DD, et al. DRG Voltage-Gated Sodium Channel 1.7 Is Upregulated in Paclitaxel-Induced Neuropathy in Rats and in Humans with Neuropathic Pain. J Neurosci. 2018;38(5):1124–36. doi: 10.1523/JNEUROSCI.0899-17.2017 29255002 PMC5792474

[10] Boehmerle W, Splittgerber U, Lazarus MB, McKenzie KM, Johnston DG, Austin DJ, et al. Paclitaxel induces calcium oscillations via an inositol 1,4,5-trisphosphate receptor and neuronal calcium sensor 1-dependent mechanism. Proc Natl Acad Sci USA. 2006;103(48):18356. doi: 10.1073/pnas.0607240103 17114292 PMC1838755

[11] Li Y, Tatsui CE, Rhines LD, North RY, Harrison DS, Cassidy RM, et al. Dorsal root ganglion neurons become hyperexcitable and increase expression of voltage-gated T-type calcium channels (Cav3.2) in paclitaxel-induced peripheral neuropathy. Pain. 2017;158(3). doi: 10.1097/j.pain.0000000000000774 27902567 PMC5303135

[12] Goshima Y, Usui H, Shiozawa T, Hida T, Kuraoka S, Takeshita S, et al. Computational analysis of the effects of antineoplastic agents on axonal transport. J Pharmacol Sci. 2010;114(2):168–79. doi: 10.1254/jphs.09352fp 20859062

[13] Flatters SJL, Bennett GJ. Studies of peripheral sensory nerves in paclitaxel-induced painful peripheral neuropathy: Evidence for mitochondrial dysfunction. Pain. 2006;122(3):245–57. doi: 10.1016/j.pain.2006.01.037 16530964 PMC1805481

[14] Rowinsky EK, Eisenhauer EA, Chaudhry V, Arbuck SG, Donehower RC. Clinical toxicities encountered with paclitaxel (Taxol). Semin Oncol. 1993;20(4 Suppl 3):1–15. 8102012

[15] Krukowski K, Eijkelkamp N, Laumet G, Hack CE, Li Y, Dougherty PM, et al. CD8+ T Cells and Endogenous IL-10 Are Required for Resolution of Chemotherapy-Induced Neuropathic Pain. J Neurosci. 2016;36(43):11074–83. doi: 10.1523/JNEUROSCI.3708-15.2016 27798187 PMC5098842

[16] Laumet G, Bavencoffe A, Edralin JD, Huo X-J, Walters ET, Dantzer R, et al. Interleukin-10 resolves pain hypersensitivity induced by cisplatin by reversing sensory neuron hyperexcitability. Pain. 2020. doi: 10.1097/j.pain.0000000000001921 32427749 PMC7962468

[17] Chen X, Zhang J, Song Y, Yang P, Yang Y, Huang Z, et al. Deficiency of anti-inflammatory cytokine IL-4 leads to neural hyperexcitability and aggravates cerebral ischemia–reperfusion injury. Acta Pharmaceutica Sinica B. 2020. doi: 10.1016/j.apsb.2020.05.002 33088684 PMC7564329

[18] Goode DJ, Whitaker EE, Mecum NE. Ovariectomy increases paclitaxel-induced mechanical hypersensitivity and reduces anti-inflammatory CD4+ T cells in the dorsal root ganglion of female mice. Journal of Neuroimmunology. 2022:577878. doi: 10.1016/j.jneuroim.2022.577878 35509138

[19] Copray JC, Mantingh I, Brouwer N, Biber K, Küst BM, Liem RS, et al. Expression of interleukin-1 beta in rat dorsal root ganglia. J Neuroimmunol. 2001;118(2):203–11. doi: 10.1016/s0165-5728(01)00324-1 11498255

[20] Wang F, Millet I, Bottomly K, Vignery A. Calcitonin gene-related peptide inhibits interleukin 2 production by murine T lymphocytes. J Biol Chem. 1992;267(29):21052–7. 1383217

[21] Richter F, Natura G, Ebbinghaus M, von Banchet GS, Hensellek S, König C, et al. Interleukin-17 sensitizes joint nociceptors to mechanical stimuli and contributes to arthritic pain through neuronal interleukin-17 receptors in rodents. Arthritis Rheum. 2012;64(12):4125–34. doi: 10.1002/art.37695 23192794

[22] Vikman KS, Hill RH, Backström E, Robertson B, Kristensson K. Interferon-γ induces characteristics of central sensitization in spinal dorsal horn neurons in vitro. Pain. 2003;106(3):241–51. 14659507 10.1016/S0304-3959(03)00262-8

[23] Nguyen MQ, von Buchholtz LJ, Reker AN, Ryba NJP, Davidson S. Single-nucleus transcriptomic analysis of human dorsal root ganglion neurons. eLife. 2021;10:e71752. doi: 10.7554/eLife.71752 34825887 PMC8626086

[24] Tavares-Ferreira D, Shiers S, Ray PR, Wangzhou A, Jeevakumar V, Sankaranarayanan I, et al. Spatial transcriptomics of dorsal root ganglia identifies molecular signatures of human nociceptors. Sci Transl Med. 2022;14(632):eabj8186. doi: 10.1126/scitranslmed.abj8186 35171654 PMC9272153

[25] Usoskin D, Furlan A, Islam S, Abdo H, Lonnerberg P, Lou D, et al. Unbiased classification of sensory neuron types by large-scale single-cell RNA sequencing. Nat Neurosci. 2015;18(1):145–53. doi: 10.1038/nn.3881 25420068

[26] Ray P, Torck A, Quigley L, Wangzhou A, Neiman M, Rao C, et al. Comparative transcriptome profiling of the human and mouse dorsal root ganglia: an. Pain. 2018;159(7):1325–45. 29561359 10.1097/j.pain.0000000000001217 PMC6008200

[27] van Velzen M, Laman JD, KleinJan A, Poot A, Osterhaus ADME, Verjans GMGM. Neuron-Interacting Satellite Glial Cells in Human Trigeminal Ganglia Have an APC Phenotype. J Immunol. 2009;183(4):2456. 19635905 10.4049/jimmunol.0900890

[28] Vagaska B, New SEP, Alvarez-Gonzalez C, D’Acquisto F, Gomez SG, Bulstrode NW, et al. MHC-class-II are expressed in a subpopulation of human neural stem cells in vitro in an IFNγ–independent fashion and during development. Scientific Reports. 2016;6(1):24251. 27080443 10.1038/srep24251 PMC4832187

[29] Dixon JS. Changes in the fine structure of satellite cells surrounding chromatolytic neurons. The Anatomical Record. 1969;163(1):101–9. doi: 10.1002/ar.1091630112 5763130

[30] Hu P, McLachlan EM. Macrophage and lymphocyte invasion of dorsal root ganglia after peripheral nerve lesions in the rat. Neuroscience. 2002;112(1):23–38. doi: 10.1016/s0306-4522(02)00065-9 12044469

[31] Lopes DM, Denk F, McMahon SB. The Molecular Fingerprint of Dorsal Root and Trigeminal Ganglion Neurons. Frontiers in Molecular Neuroscience. 2017;10:304. doi: 10.3389/fnmol.2017.00304 29018326 PMC5623188

[32] Pfannenstiel LW, Lam SS, Emens LA, Jaffee EM, Armstrong TD. Paclitaxel enhances early dendritic cell maturation and function through TLR4 signaling in mice. Cell Immunol. 2010;263(1):79–87. doi: 10.1016/j.cellimm.2010.03.001 20346445 PMC2862830

[33] Cella M, Engering A, Pinet V, Pieters J, Lanzavecchia A. Inflammatory stimuli induce accumulation of MHC class II complexes on dendritic cells. Nature. 1997;388(6644):782–7. doi: 10.1038/42030 9285591

[34] Bosch B, Heipertz EL, Drake JR, Roche PA. Major histocompatibility complex (MHC) class II-peptide complexes arrive at the plasma membrane in cholesterol-rich microclusters. J Biol Chem. 2013;288(19):13236–42. doi: 10.1074/jbc.M112.442640 23532855 PMC3650363

[35] Zhang H, Dougherty PM. Enhanced excitability of primary sensory neurons and altered gene expression of neuronal ion channels in dorsal root ganglion in paclitaxel-induced peripheral neuropathy. Anesthesiology. 2014;120(6):1463–75. doi: 10.1097/ALN.0000000000000176 24534904 PMC4031279

[36] Siau C, Xiao W, Bennett GJ. Paclitaxel- and vincristine-evoked painful peripheral neuropathies: loss of epidermal innervation and activation of Langerhans cells. Exp Neurol. 2006;201(2):507–14. doi: 10.1016/j.expneurol.2006.05.007 16797537 PMC1805691

[37] Patil MJ, Hovhannisyan AH, Akopian AN. Characteristics of sensory neuronal groups in CGRP-cre-ER reporter mice: Comparison to Nav1.8-cre, TRPV1-cre and TRPV1-GFP mouse lines. PLOS ONE. 2018;13(6):e0198601–e. doi: 10.1371/journal.pone.0198601 29864146 PMC5986144

[38] Goswami SC, Mishra SK, Maric D, Kaszas K, Gonnella GL, Clokie SJ, et al. Molecular signatures of mouse TRPV1-lineage neurons revealed by RNA-Seq transcriptome analysis. J Pain. 2014;15(12):1338–59. doi: 10.1016/j.jpain.2014.09.010 25281809 PMC5469214

[39] Ho C, Zhao J, Malinowski S, Chahine M, O’Leary ME. Differential Expression of Sodium Channel β Subunits in Dorsal Root Ganglion Sensory Neurons*. Journal of Biological Chemistry. 2012;287(18):15044–53. 22408255 10.1074/jbc.M111.333740 PMC3340221

[40] Luo X, Huh Y, Bang S, He Q, Zhang L, Matsuda M, et al. Macrophage Toll-like Receptor 9 Contributes to Chemotherapy-Induced Neuropathic Pain in Male Mice. The Journal of Neuroscience. 2019;39(35):6848–64. doi: 10.1523/JNEUROSCI.3257-18.2019 31270160 PMC6733562

[41] Polomano RC, Mannes AJ, Clark US, Bennett GJ. A painful peripheral neuropathy in the rat produced by the chemotherapeutic drug, paclitaxel. Pain. 2001;94(3):293–304. doi: 10.1016/S0304-3959(01)00363-3 11731066

[42] Smith SB, Crager SE, Mogil JS. Paclitaxel-induced neuropathic hypersensitivity in mice: responses in 10 inbred mouse strains. Life Sci. 2004;74(21):2593–604. doi: 10.1016/j.lfs.2004.01.002 15041441

[43] Shi Q, Cai X, Shi G, Lv X, Yu J, Wang F. Interleukin-4 protects from chemotherapy-induced peripheral neuropathy in mice modal via the stimulation of IL-4/STAT6 signaling. Acta Cir Bras. 2018;33(6):491–8. doi: 10.1590/s0102-865020180060000003 30020310

[44] Saxena A, Khosraviani S, Noel S, Mohan D, Donner T, Hamad AR. Interleukin-10 paradox: A potent immunoregulatory cytokine that has been difficult to harness for immunotherapy. Cytokine. 2015;74(1):27–34. doi: 10.1016/j.cyto.2014.10.031 25481648 PMC4454631

[45] Lech-Maranda E, Bienvenu J, Michallet AS, Houot R, Robak T, Coiffier B, et al. Elevated IL-10 plasma levels correlate with poor prognosis in diffuse large B-cell lymphoma. Eur Cytokine Netw. 2006;17(1):60–6. 16613764

[46] Nelson DR, Tu Z, Soldevila-Pico C, Abdelmalek M, Zhu H, Xu YL, et al. Long-term interleukin 10 therapy in chronic hepatitis C patients has a proviral and anti-inflammatory effect. Hepatology. 2003;38(4):859–68. doi: 10.1053/jhep.2003.50427 14512873

[47] Banchereau J, Steinman RM. Dendritic cells and the control of immunity. Nature. 1998;392(6673):245–52. doi: 10.1038/32588 9521319

[48] Harding CV, Unanue ER. Quantitation of antigen-presenting cell MHC class II/peptide complexes necessary for T-cell stimulation. Nature. 1990;346(6284):574–6. doi: 10.1038/346574a0 2115981

[49] Francisco Junior RDS, Temerozo JR, Ferreira CDS, Martins Y, Souza TML, Medina-Acosta E, et al. Differential haplotype expression in class I MHC genes during SARS-CoV-2 infection of human lung cell lines. Front Immunol. 2022;13:1101526. doi: 10.3389/fimmu.2022.1101526 36818472 PMC9929942

[50] Ivashkiv LB. IFNγ: signalling, epigenetics and roles in immunity, metabolism, disease and cancer immunotherapy. Nat Rev Immunol. 2018;18(9):545–58. 29921905 10.1038/s41577-018-0029-z PMC6340644

[51] Rathinam VA, Appledorn DM, Hoag KA, Amalfitano A, Mansfield LS. Campylobacter jejuni-induced activation of dendritic cells involves cooperative signaling through Toll-like receptor 4 (TLR4)-MyD88 and TLR4-TRIF axes. Infect Immun. 2009;77(6):2499–507. doi: 10.1128/IAI.01562-08 19332531 PMC2687361

[52] Michelsen KS, Aicher A, Mohaupt M, Hartung T, Dimmeler S, Kirschning CJ, et al. The role of toll-like receptors (TLRs) in bacteria-induced maturation of murine dendritic cells (DCS). Peptidoglycan and lipoteichoic acid are inducers of DC maturation and require TLR2. J Biol Chem. 2001;276(28):25680–6. doi: 10.1074/jbc.M011615200 11316801

[53] Wang TT, Xu XY, Lin W, Hu DD, Shi W, Jia X, et al. Activation of Different Heterodimers of TLR2 Distinctly Mediates Pain and Itch. Neuroscience. 2020;429:245–55. doi: 10.1016/j.neuroscience.2020.01.010 31954829

[54] Cameron JS, Alexopoulou L, Sloane JA, DiBernardo AB, Ma Y, Kosaras B, et al. Toll-like receptor 3 is a potent negative regulator of axonal growth in mammals. J Neurosci. 2007;27(47):13033–41. doi: 10.1523/JNEUROSCI.4290-06.2007 18032677 PMC4313565

[55] Barajon I, Serrao G, Arnaboldi F, Opizzi E, Ripamonti G, Balsari A, et al. Toll-like receptors 3, 4, and 7 are expressed in the enteric nervous system and dorsal root ganglia. J Histochem Cytochem. 2009;57(11):1013–23. doi: 10.1369/jhc.2009.953539 19546475 PMC2762881

[56] Xu ZZ, Kim YH, Bang S, Zhang Y, Berta T, Wang F, et al. Inhibition of mechanical allodynia in neuropathic pain by TLR5-mediated A-fiber blockade. Nat Med. 2015;21(11):1326–31. doi: 10.1038/nm.3978 26479925 PMC4752254

[57] Zhang ZJ, Guo JS, Li SS, Wu XB, Cao DL, Jiang BC, et al. TLR8 and its endogenous ligand miR-21 contribute to neuropathic pain in murine DRG. J Exp Med. 2018;215(12):3019–37. doi: 10.1084/jem.20180800 30455267 PMC6279408

[58] Szabo-Pardi TA, Barron LR, Lenert ME, Burton MD. Sensory Neuron TLR4 mediates the development of nerve-injury induced mechanical hypersensitivity in female mice. Brain Behav Immun. 2021;97:42–60. doi: 10.1016/j.bbi.2021.06.011 34174335 PMC8453057

[59] Baumgart M, Moos V, Schuhbauer D, Müller B. Differential expression of major histocompatibility complex class II genes on murine macrophages associated with T cell cytokine profile and protective/suppressive effects. Proc Natl Acad Sci U S A. 1998;95(12):6936–40. doi: 10.1073/pnas.95.12.6936 9618517 PMC22692

[60] Kivisäkk P, Mahad DJ, Callahan MK, Trebst C, Tucky B, Wei T, et al. Human cerebrospinal fluid central memory CD4+ T cells: evidence for trafficking through choroid plexus and meninges via P-selectin. Proceedings of the National Academy of Sciences of the United States of America. 2003;100(14):8389–94. doi: 10.1073/pnas.1433000100 12829791 PMC166239

[61] Axtell RC, Steinman L. Gaining entry to an uninflamed brain. Nat Immunol. 2009;10(5):453–5. doi: 10.1038/ni0509-453 19381137

[62] Liu X-J, Zhang Y, Liu T, Xu Z-Z, Park C-K, Berta T, et al. Nociceptive neurons regulate innate and adaptive immunity and neuropathic pain through MyD88 adapter. Cell Res. 2014;24(11):1374–7. doi: 10.1038/cr.2014.106 25112711 PMC4220153

[63] Iwai H, Ataka K, Suzuki H, Dhar A, Kuramoto E, Yamanaka A, et al. Tissue-resident M2 macrophages directly contact primary sensory neurons in the sensory ganglia after nerve injury. J Neuroinflammation. 2021;18(1):227. doi: 10.1186/s12974-021-02283-z 34645458 PMC8513227

[64] Singh SK, Krukowski K, Laumet GO, Weis D, Alexander JF, Heijnen CJ, et al. CD8+ T cell-derived IL-13 increases macrophage IL-10 to resolve neuropathic pain. JCI Insight. 2022;7(5). doi: 10.1172/jci.insight.154194 35260535 PMC8983134

[65] Schindelin J, Arganda-Carreras I, Frise E, Kaynig V, Longair M, Pietzsch T, et al. Fiji: an open-source platform for biological-image analysis. Nat Methods. 2012;9(7):676–82. doi: 10.1038/nmeth.2019 22743772 PMC3855844

