# Supplementary material for: Expression of MHC II in DRG neurons attenuates paclitaxel-induced cold hypersensitivity in male and female mice
Source: PLoS One. 2024 Feb 8;19(2):e0298396. doi: 10.1371/journal.pone.0298396 (PMC10852343; doi:10.1371/journal.pone.0298396)
Supplement: S1 File — (PDF) [file pone.0298396.s001.pdf]

Supplementary Materials

Supplemental Tables

Group	Dunnett's multiple comparisons test	Summary	P Value
cHET Female Naive	1 vs. 4	*	0.0127
WT Female Naive	1 vs. 2	**	0.0019
	1 vs. 3	*	0.0154
	1 vs. 4	*	0.0115
cHET Male Naive	1 vs. 3	***	0.0005
	1 vs. 4	****	<0.0001
WT Male Naive	1 vs. 2	*	0.0198
	1 vs. 3	*	0.0235

S1 Table. Repeated measures mixed-effects model (REML) with Dunnett's multiple comparison test for naive female and male WT and cHET for TPP test shown in **Fig 8A, B**.

Group	Dunnett's multiple comparisons test	Summary	P Value
cHET Female PTX	1 vs. 2	****	<0.0001
	1 vs. 3	****	<0.0001
	1 vs. 4	****	<0.0001
WT Female PTX	1 vs. 2	***	0.0003
	1 vs. 3	***	0.0001
	1 vs. 4	****	<0.0001
cHET Male PTX	1 vs. 2	***	0.0005
	1 vs. 3	****	<0.0001
	1 vs. 4	****	<0.0001
WT Male PTX	1 vs. 2	*	0.0167
	1 vs. 3	***	0.0005
	1 vs. 4	****	<0.0001

S2 Table. Repeated measures mixed-effects model (REML) with Dunnett's multiple comparison test for PTX-treated female and male WT and cHET for TPP test shown in **Fig 8E, F**.

Strain	Common Name	Strain No.
C57BL6/J	B6	#000664
B6.129X1-$H2\text{-}Ab1^{tm1Koni}$/J	$\text{MHCII}^{fl/fl}$	#013181
B6.129-$Trpv1^{tm1(cre)Bbm}$/J	TRPV1^{Cre} progenitor line	#017769
B6.Cg-$Gt(ROSA)26Sor^{tm14(CAG\text{-}tdTomato)Hze}$/J	td-Tomato reporter line	#007914

S3 Table. Jackson Laboratory mouse strains used in the study.

Gene	Primer Name	Sequence
MHCII	common forward	5'-CTC TAC ACC CCC AAC ACA CC-3'
	wildtype reverse	5'-AGT GAG CGA GCA CAG ACA AG-3'
	floxed reverse	5'-TCG CCT TCT TGA CGA GTT CT-3'
CRE	Forward	5'-TTC CCG CAG AAC CTG AAG ATG-3'
	Reverse	5'-CCC CAG AAA TGC CAG ATT ACG-3'

S4 Table. Primer sequences for TRPV-1 lineage MHCII conditional knockout mice.

Antibody/Stain	Vendor	Clone	RRID	Dilution (application)
Armenian hamster 488	JIR	polyclonal	AB_2338996	1:400 (IHC)
Beta-tubulin	Abcam	polyclonal	AB_869991	1:8000 (western)
CD3	BioLegend	145-2C11	AB_312667	1:100 (IHC)
CD3 eFluor506	ThermoFisher	17A2	AB_2637122	1 µl/test (FC)
CD4	Bio Xcell	GK1.5	AB_1107636	2 µg/ml (IHC)
CD4 PE-Texas Red	ThermoFisher	RM4-5	AB_10373812	1 µl/test (FC)
CD45 488	BioLegend	S18009D	AB_2888853	0.5 µl/test (FC)
CD8 Super Bright 645	ThermoFisher	53-6.7	AB_2662353	1.25 µl/test (FC)
CD11b 488	Biolegend	M1/70	AB_389305	1:100 (IHC)
CD11c 488	Biolegend	N418	AB_389306	1:100 (IHC)
FABP7	Invitrogen	Polyclonal	AB_2542449	1:5000 (IHC)
FoxP3, PerCP-Cyanine5.5	ThermoFisher	FJK-16s	AB_914351	0.625 µl/test (FC)
GLAST	Miltenyi	ACSA-1	AB_2811532	1:50 (IHC)
IL-4, PE-Cyanine7	ThermoFisher	11B11	AB_2573520	0.625 µl/test (FC)
IL-10, Alexa Fluor 700	ThermoFisher	JES5-16E3	AB_891568	0.625 µl/test (FC)
LAP, PerCP-eFluor 710	ThermoFisher	TW7-16B4	AB_10853808	0.625 µl/test (FC)
LIVE/DEAD™ Fixable Violet Dead Cell Stain	ThermoFisher	NA	NA	0.2 µl/test (FC)
MHC Class II, I-A/I-E	BioLegend	M5/114.15.2	AB_313329 (APC)<br>AB_493525 (647)<br>AB_493523 (488)	1:2500 (IHC, ICC); 1:100 (FC); 3 µg (western)
NK1.1, Super Bright 600	ThermoFisher	PK136	AB_2637451	2.5 µl/test (FC)
Rat IgG2b kappa	eBioscience (APC)<br>Biolegend (AF647)	polyclonal	AB_470176 (APC)<br>AB_389343 (AF647)	1:2500 (IHC, ICC); 1:100 (FC)
Rabbit DyLight405	JIR	polyclonal	AB_2340616	1:200 (IHC)
Rat Cy-3	JIR	polyclonal	AB_2340667	1:200 (IHC)

Rabbit AlexaFluor488	JIR	polyclonal	AB_2313584	1:200 (IHC, ICC)
Rabbit 647	JIR	polyclonal	AB_2492288	1:200 (IHC); 1:2000 (western)
UCHL1/PGP9.5	Proteintech	polyclonal	AB_2210497	1:12500 (IHC); 1:15000 (ICC); 1:114 (FC)

S5 Table. Antibody/Stain details for each application. JIR= Jackson ImmunoResearch, NA= Not Applicable.

Supplemental Figures

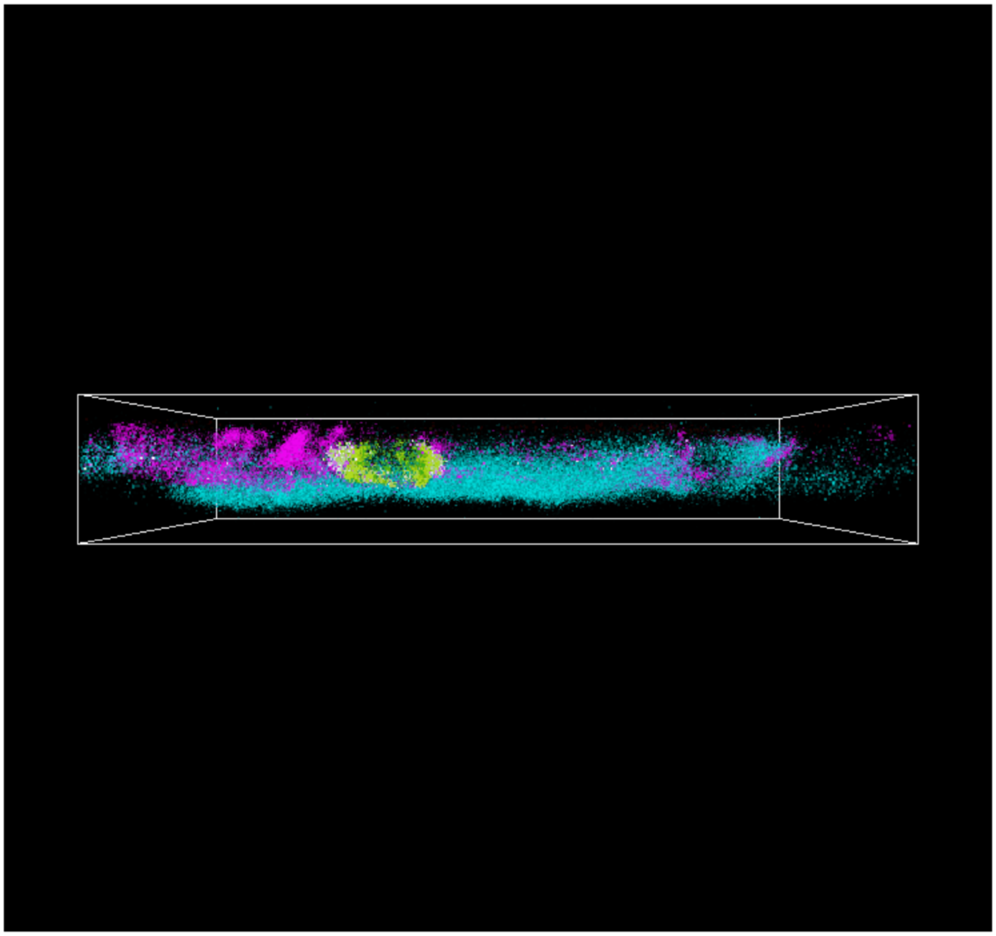

S1 Fig. CD4$^+$ T cell breaches the SGC barrier in female mouse DRG tissue. 3D projection of confocal Z-slice images of a CD4$^+$ T cell in close proximity to a DRG neuron from **Fig 1C, D.** 3D volume rotated around the X-axis at a rate of 10 frames/second (see **S1_Video)**.

A. Day 14 PTX

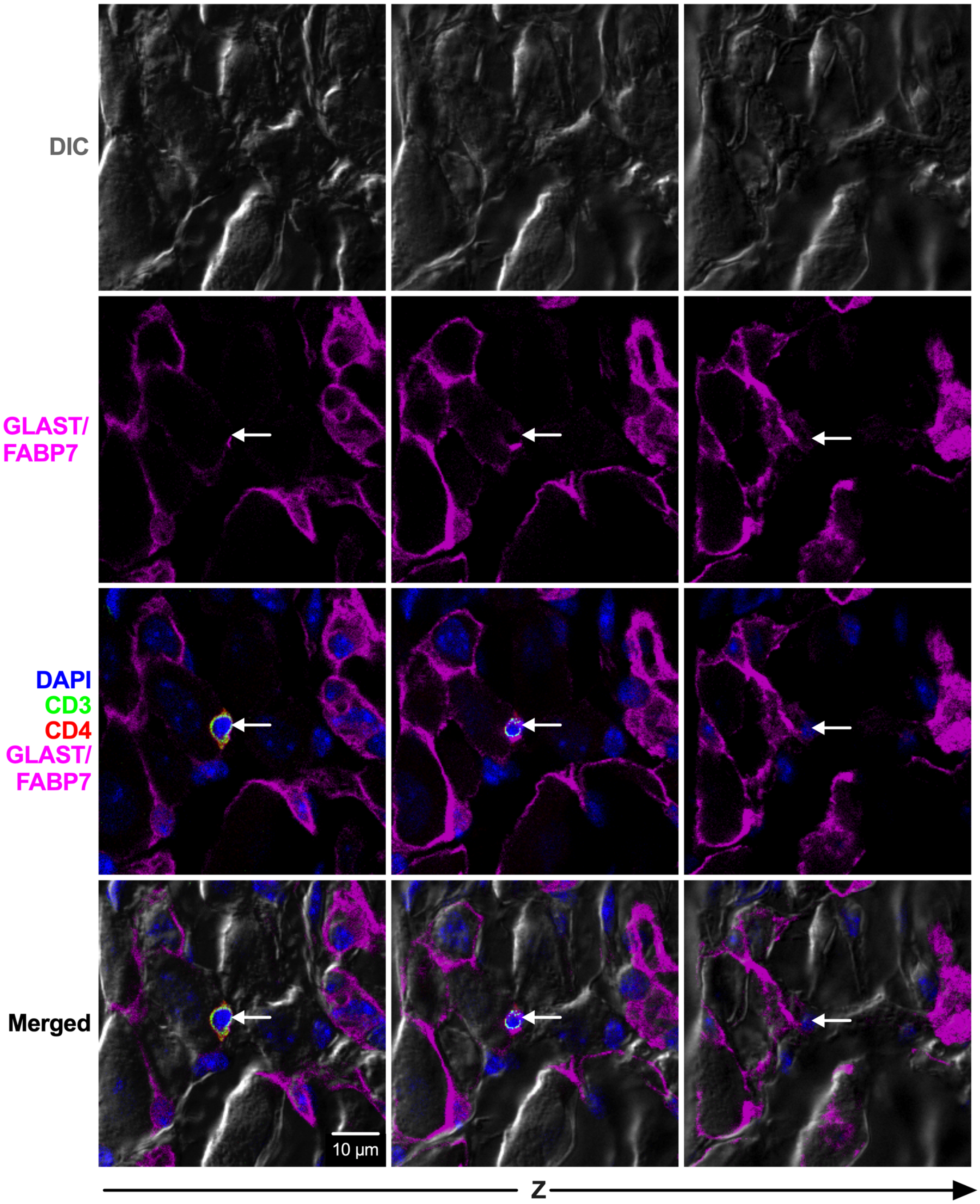

B. Day 14 PTX

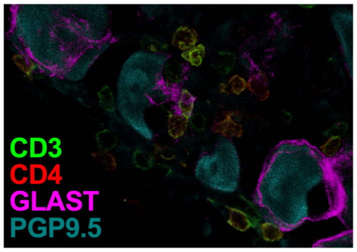

S2 Fig. CD4$^+$ T cells are in close proximity to neurons in female mouse DRG tissue. (A) Differential inference contrast (DIC), fluorescence, and merged confocal Z-slice images of a CD4$^+$ T cell in close proximity to a neuron in female mouse DRG tissue. **(B)** Hotspots of CD4$^+$ T cells in the DRG of a day 14 PTX-treated female mouse.

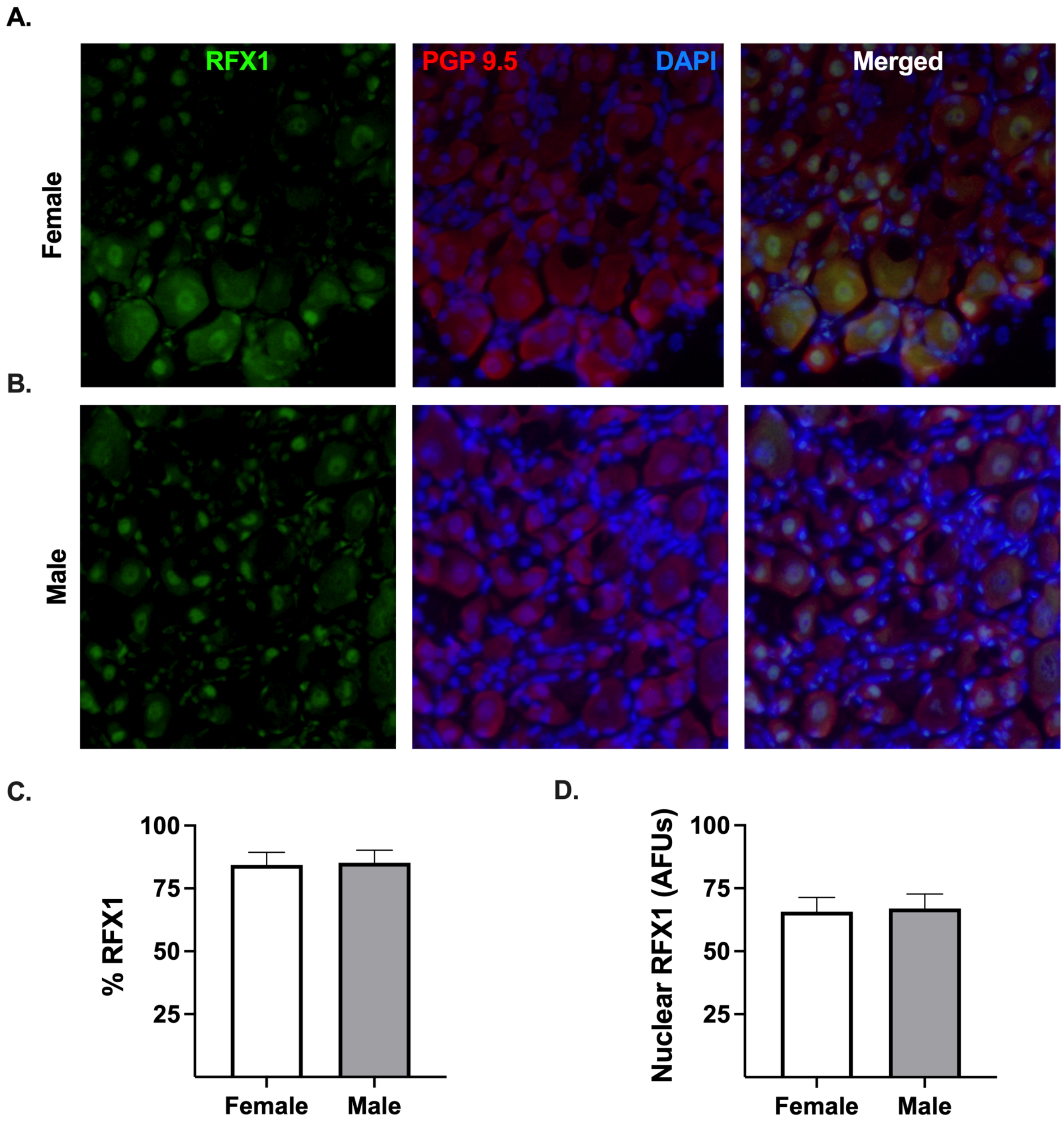

S3 Fig. DRG neurons from naive female and male mice express nuclear RFX1 protein. (**A**, **B**) Immunohistochemistry of RFX1 (green) staining in the nucleus (DAPI, blue) and cytoplasm of DRG neurons (PGP9.5^{+}, red) from naïve female (**A**) and male (**B**) mice. (**C**) Percent of RFX1^{+} DRG neurons and (**D**) nuclear neuron RFX1 intensity (AFUs) for naïve female (white bar) and male (gray bar) mice. Statistical analysis by unpaired t-test, n=8.

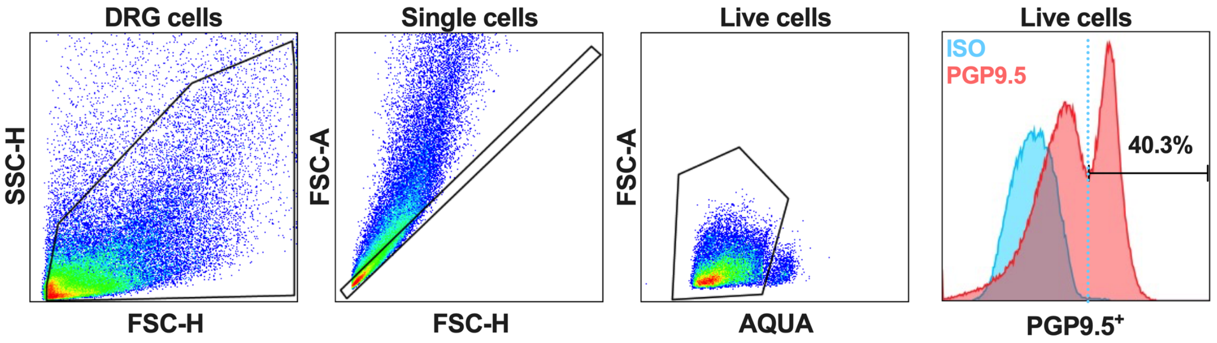

S4 Fig. PGP9.5⁺ neurons comprise a fraction of total DRG cells. A nested gating strategy was used to identify PGP9.5⁺ neurons from total DRG cells. Representative histogram overlay of PGP9.5 (red histogram) and Rat IgG2b kappa Isotype Control (ISO; blue histogram with dashed line representing the negative/positive cutoff).

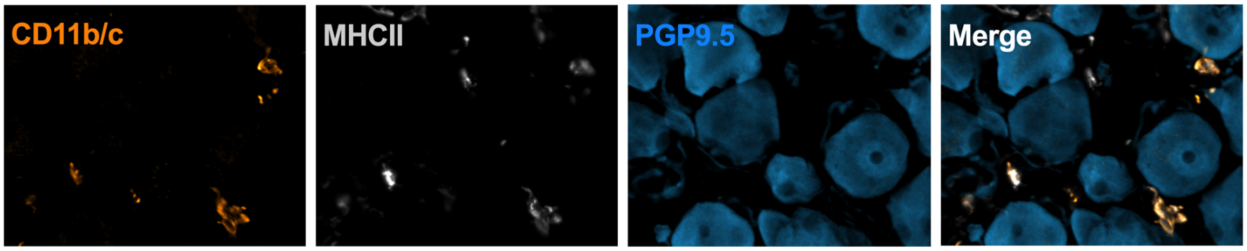

S5 Fig. CD11b/c⁺ immune cells in DRG tissue express MHCII. IHC staining of CD11b and/or CD11c (orange), MHCII (gray), and PGP9.5 (blue) in naïve male L4 DRG tissue. Representative single channel and merged widefield fluorescence images of immune cells in DRG tissue co-stained with CD11b/c and MHCII.

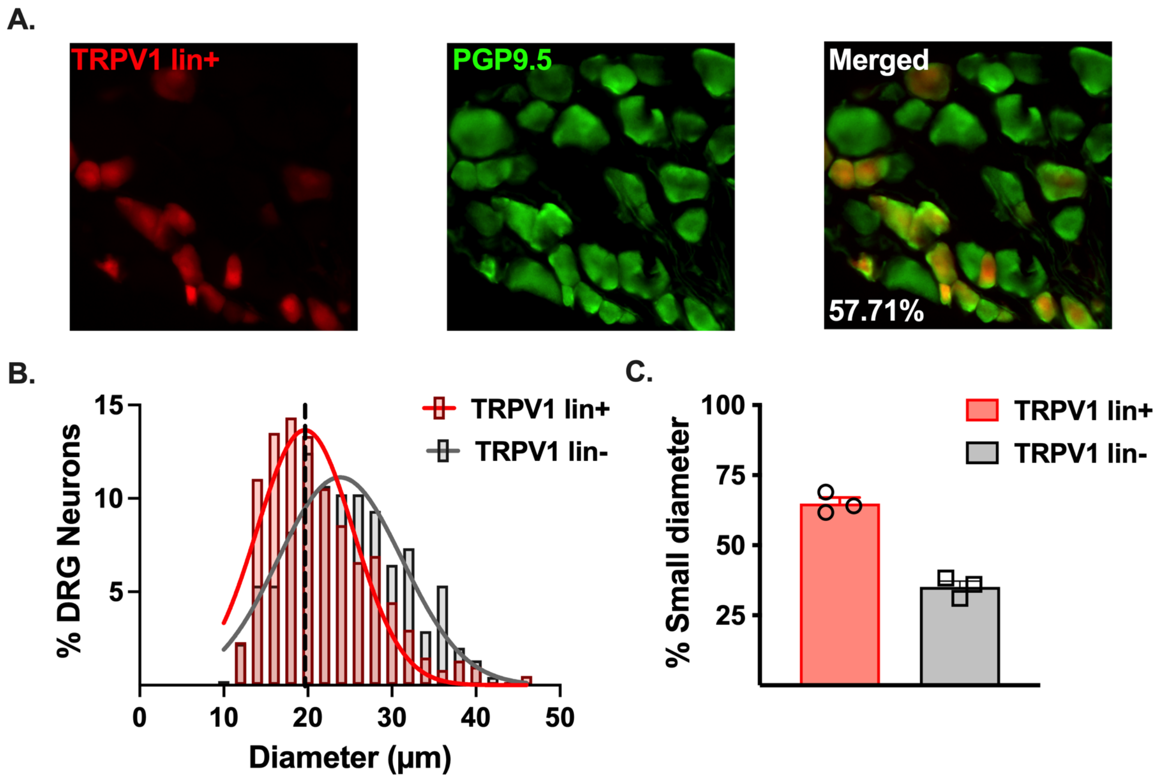

S6 Fig. High throughput automated image analysis module identifies small and large diameter neurons in female DRG tissue. (**A**) Representative image of td-Tomato-reporter in TRPV1-lineage (red) neurons in naïve female DRG. Percent of TRPV1-lineage neurons from total PGP9.5^{+} (green) neurons shown in merged. (**B**) Gaussian distribution of the diameter (µm) of TRPV1-lineage (red) and non-TRPV1-lineage (gray) neurons. Black dashed line: mode of diameter of TRPV1-lineage neurons (19.66 µm). **(C)** Percent of small diameter TRPV1-lineage positive (red bar) and negative (gray bar) neurons, n=3.

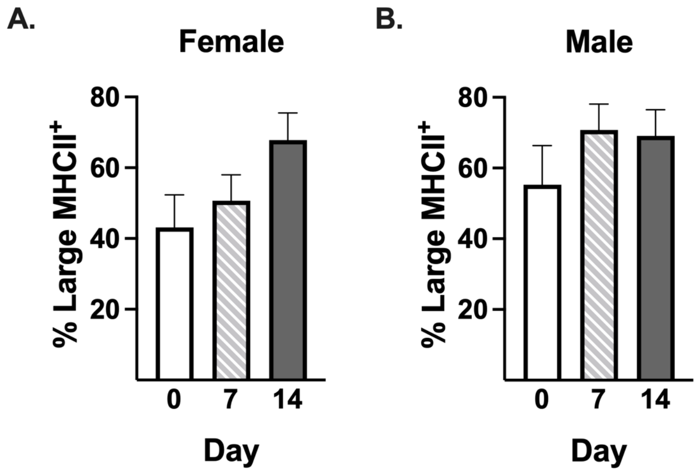

S7 Fig. PTX does not change the percent of large diameter neurons that express MHCII in male and female DRG. Percent of large diameter (≥30 µm) neurons positive for MHCII in female (**A**) and male (**B**) DRG before (day 0) and after PTX treatment (day 7 and 14), n=8.
