# Supplementary material for: Expression of MHC II in DRG neurons attenuates paclitaxel-induced cold hypersensitivity in male and female mice
Source: PLoS One. 2024 Feb 8;19(2):e0298396. doi: 10.1371/journal.pone.0298396 (PMC10852343; doi:10.1371/journal.pone.0298396)
Supplement: S1 Raw images — (PDF) [file pone.0298396.s003.pdf]

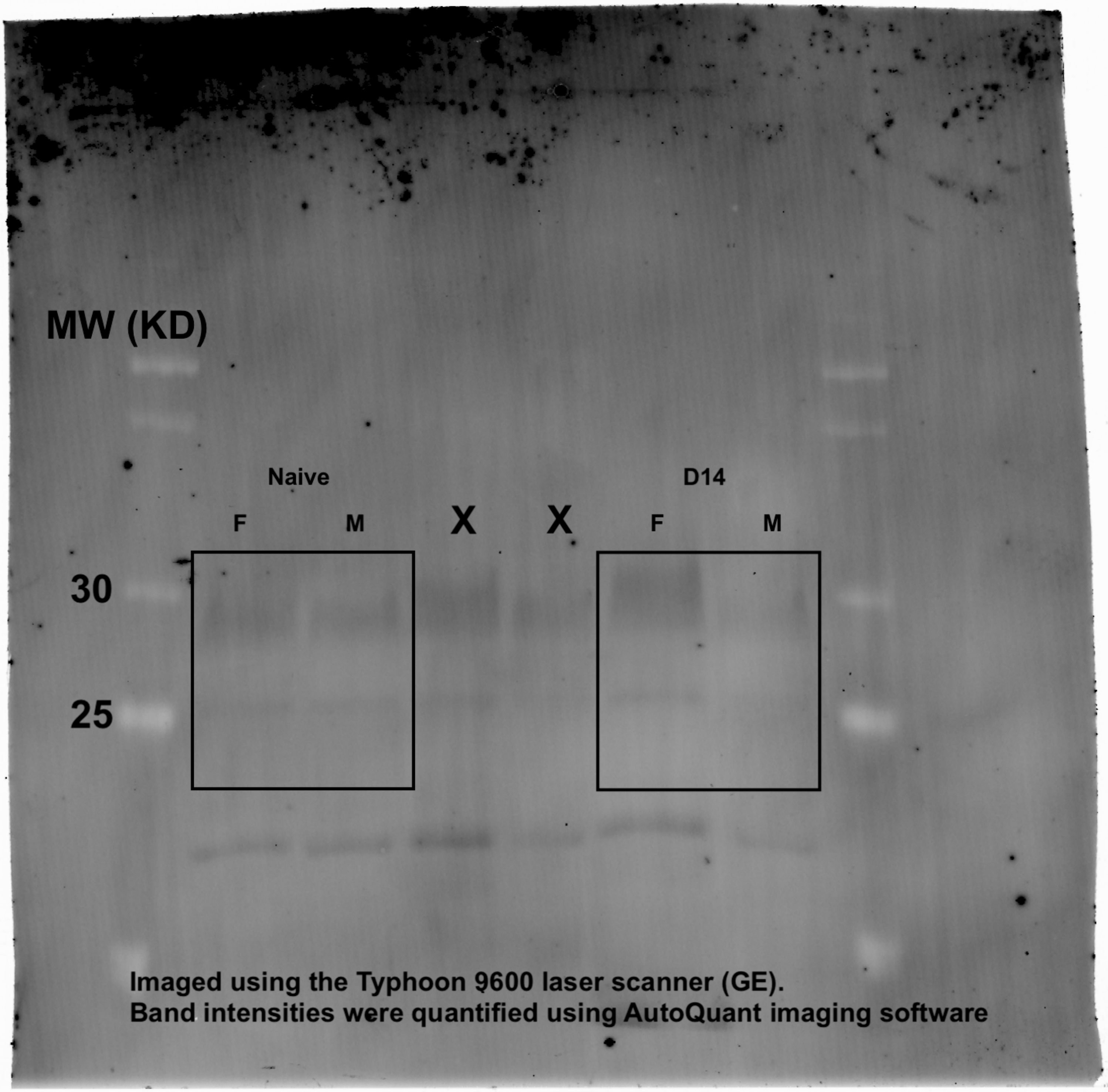
MW (KD)
Naive
D14
F
M
X
X
F
M
30
25
Imaged using the Typhoon 9600 laser scanner (GE).
Band intensities were quantified using AutoQuant imaging software

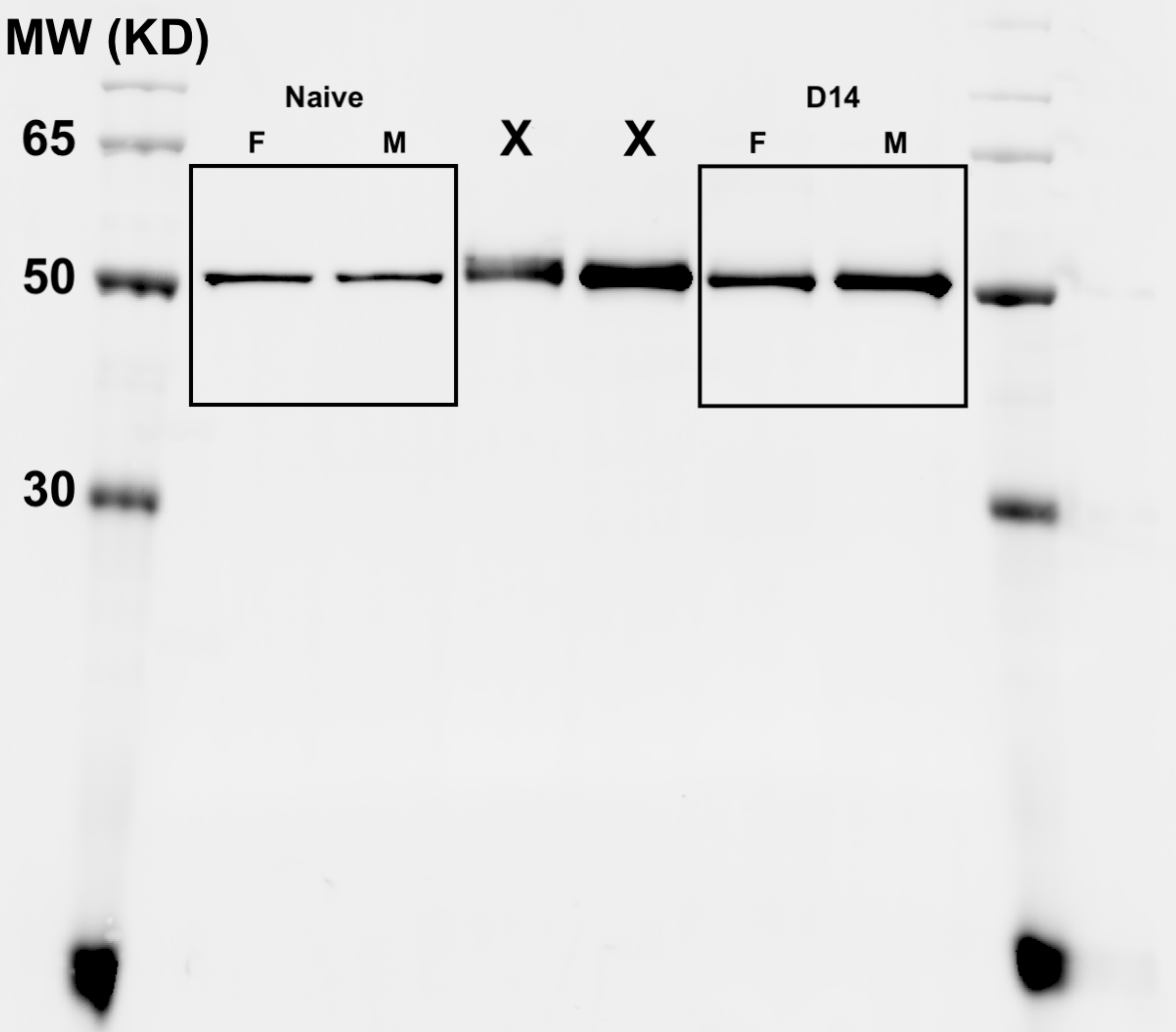

Imaged using the Typhoon 9600 laser scanner (GE).
Band intensities were quantified using AutoQuant imaging software
