# Supplementary figures and images for: Expression of MHC II in DRG neurons attenuates paclitaxel-induced cold hypersensitivity in male and female mice
Source: PLoS One. 2024 Feb 8;19(2):e0298396. doi: 10.1371/journal.pone.0298396 (PMC10852343; doi:10.1371/journal.pone.0298396)

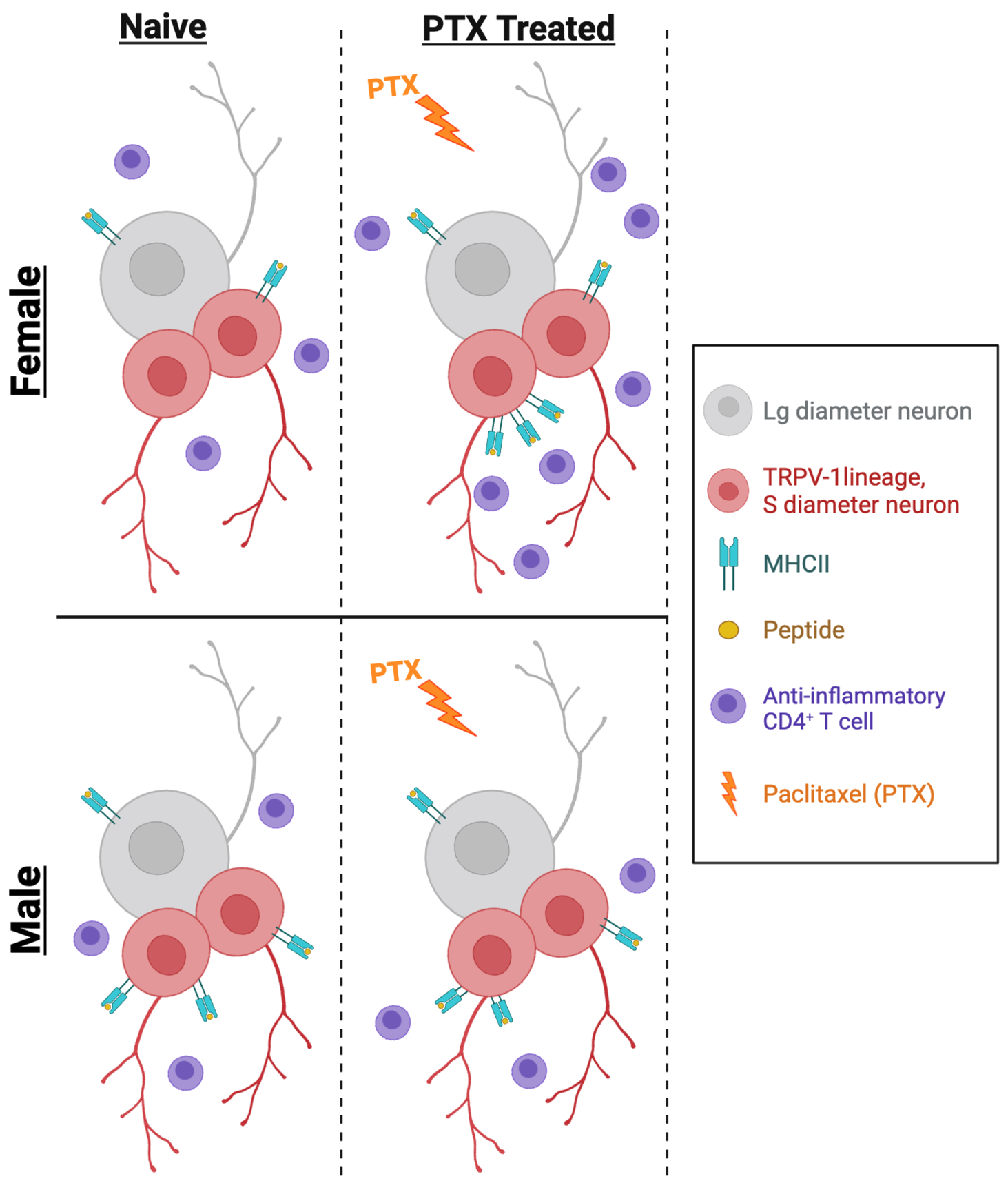

Supplement: S1 Graphical abstract — (TIFF) [file pone.0298396.s005.tiff]
